# Polydopamine‐Coated Selenium Nanoparticles as a Stable Catalyst for Tunable and Sustained Nitric Oxide Generation

**DOI:** 10.1002/smsc.202500151

**Published:** 2025-06-01

**Authors:** Shu Geng, Qingqing Fan, Kang Lin, Federico Mazur, Rona Chandrawati

**Affiliations:** ^1^ School of Chemical Engineering and Australian Centre for Nanomedicine (ACN) The University of New South Wales (UNSW Sydney) Sydney NSW 2052 Australia

**Keywords:** catalysis, core‐shell nanoparticle, nitric oxide, polydopamine coating, selenium nanoparticles

## Abstract

Nitric oxide (NO) is a therapeutic gas molecule involved in numerous physiological and pathological processes. However, its clinical application is limited by its short half‐life and limited diffusion distance in human tissues, necessitating the development of effective NO delivery strategies. *In situ* NO generation via catalytic decomposition of endogenous NO donors has emerged as a promising approach. Selenium nanoparticles (SeNPs) have demonstrated high catalytic efficiency for NO generation with low cytotoxicity, but their performance is hindered by poor stability under physiological conditions and pH‐dependent activity. To address these limitations, in this study, selenium‐polydopamine core–shell nanoparticles (Se@PDA NPs) are developed to improve catalytic stability and mitigate pH sensitivity. The PDA coating enables consistent NO delivery across a broad pH range (5.5–8.5), expanding their therapeutic potential. NO generation is tunable by varying the PDA coating thickness, and the nanoparticles exhibit excellent biocompatibility and enhanced cellular uptake. In human coronary artery smooth muscle cells, Se@PDA NPs catalyze intracellular NO generation from endogenous *S*‐nitrosothiols and promote the formation of multicellular aggregates, indicating potential activation of intercellular communication. The Se@PDA NPs maintain sustained NO generation over five doses and remain active for at least two months, demonstrating strong potential for NO‐based therapies.

## Graphical abstract

Polydopamine‐coated selenium nanoparticles (Se@PDA NPs) are effective catalysts for *in situ* NO generation. The PDA coating enhances cellular uptake, allowing for intracellular NO generation from endogenous NO donors, such as *S*‐nitrosoglutathione. Se@PDA NPs exhibit high biocompatibility, sustain NO generation over five doses, and remain active for at least 2 months, demonstrating strong potential for NO‐based therapeutic applications.

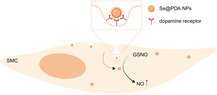

## 1 Introduction

Nitric oxide (NO) is a therapeutic gas molecule that plays a critical role in numerous physiological and pathological processes, such as regulating vascular homeostasis,^[^ ^1^ ^]^ retinal function,^[^ ^2^ ^]^ and immune responses,^[^ ^3^ ^]^ as well as in wound healing,^[^ ^4^ ^]^ anticancer,^[^ ^5^ ^]^ and antimicrobial^[^ ^6^ ^]^ activities. Notably, the biological effects of NO are concentration‐dependent, prompting cell survival and proliferation at lower concentrations (nM), and inducing cell apoptosis as well as exhibiting antibacterial or anticancer properties at higher concentrations (μM).^[^ ^7^ ^]^ Therefore, delivering NO in a tunable and controllable manner is of great importance.

NO delivery can be categorized into non‐catalytic and catalytic approaches. The non‐catalytic approach relies on the encapsulation^[^ ^8^ ^]^ or modification^[^ ^9^ ^]^ of NO donors to a delivery vehicle, enabling targeted and controlled NO release on‐site. While effective, this approach is limited by its payload capacity, an important issue for chronic diseases that require sustained NO levels at the target site for long periods.^[^ ^10^ ^]^ On the other hand, the catalytic approach involves using activated materials,^[^ ^11^ ^]^ coatings,^[^ ^12^ ^]^ or nanomaterials^[^ ^13^ ^]^ to catalytically decompose endogenous NO donors, facilitating *in situ* NO generation that continues as long as these donors are available. Several materials have demonstrated this property, including copper,^[^ ^14^ ^]^ zinc oxide,^[^ ^15^, ^16^ ^]^ and ceria nanoparticles,^[^ ^17^ ^]^ copper‐doped metal‐organic frameworks,^[^ ^18^ ^]^ and organoselenium compounds.^[^ ^19^, ^20^ ^]^ We recently demonstrated that selenium nanoparticles (SeNPs) are highly effective catalysts for NO generation from both endogenous and exogenous NO donors, producing 7.5 μM of NO within 30 min using as little as 0.1 μg mL^−1^ of SeNPs.^[^ ^21^ ^]^ However, prolonged exposure of SeNPs to NO donors under physiological conditions led to a reduction in catalytic activity. This was attributed to particle aggregation and the transition from an amorphous to a crystalline structure. To address this limitation, we investigated polydopamine (PDA), a self‐adherent polymer,^[^ ^22^ ^]^ as a surface modification for SeNPs to enhance their stability and maintain catalytic efficiency.

PDA offers numerous advantages, including high biocompatibility,^[^ ^23^ ^]^ biodegradability,^[^ ^24^ ^]^ antibacterial properties,^[^ ^25^ ^]^ and high affinity for diverse materials.^[^ ^26^ ^]^ These properties, compounded by a simple coating process, have led to extensive studies on PDA‐coated nanomaterials, showing promising potential across a wide range of applications.^[^ ^27^, ^28^, ^29^ ^]^ For instance, PDA‐coated SeNPs (Se@PDA NPs) have been developed to protect cell components against oxidative damage.^[^ ^30^ ^]^ Specifically, SeNPs were chosen due to their well‐known ability to mimic the enzyme glutathione peroxidase (GPx), an essential antioxidant enzyme that protects cells from oxidative damage by reducing harmful reactive oxygen species (ROS). Similarly, PDA was included due to its ability to mimic non‐enzymatic antioxidant biomolecules that protect cells from oxidative stress. By combining both materials, the researchers created a nanocomposite that not only mimics the enzymatic antioxidant activity of GPx but also integrates the broad‐spectrum ROS scavenging capabilities of non‐enzymatic antioxidants. Alternatively, Se@PDA nanocomposites loaded with indocyanine green (ICG) have been developed for antibacterial applications.^[^ ^31^ ^]^ The PDA coating endowed the nanocomposite with excellent photothermal properties, enabling the conversion of near‐infrared (NIR) light into heat, while also improving the biocompatibility and chemical reactivity. ICG, an antibacterial drug, absorbed NIR light to produce ROS, making it highly effective in disrupting bacterial cells and biofilms. This combination significantly enhanced the platform's bactericidal properties, particularly against pathogens like *Staphylococcus aureus* and *Escherichia coli*, rapidly removing bacteria from wounds *in vivo* and promoting wound healing without toxic effects. Moreover, a recent study demonstrated that PDA coatings can enhance cellular uptake by facilitating the binding of catechol and amine groups of PDA to the D_2_ dopamine receptors (DRD2) on cells.^[^ ^32^ ^]^ This finding highlights the potential of PDA‐based nanomedicines, specifically in targeting specific cell types. Additionally, the chelation ability attributed to the catechol and amine groups of PDA facilitates the formation of metal‐PDA networks. These include zinc‐coordinated PDA surfaces for antibacterial applications^[^ ^33^ ^]^ or copper‐coordinated PDA surfaces for NO generation and antithrombotic applications,^[^ ^34^ ^]^ amongst others.^[^ ^35^, ^36^ ^]^ Furthermore, PDA coatings have also been shown to prevent particle aggregation, enhancing catalytic activity.^[^ ^37^ ^]^

Herein, we synthesized Se@PDA NPs with varying PDA coating thicknesses and confirmed their core–shell structures through morphological and structural characterization. We then evaluated the pH‐dependent catalytic activity of SeNPs through both real‐time and cumulative NO generation, while also exploring the ability to tune NO generation by varying the PDA coating thickness. Additionally, the stability of Se@PDA NPs under physiological conditions and long‐term storage was examined. Lastly, we assessed the biocompatibility and cellular uptake of the Se@PDA NPs using mouse embryonic fibroblasts and human coronary artery smooth muscle cells (HCASMCs). Collectively, we present a stable and reusable NO delivery platform utilizing Se@PDA NPs, promoting their potential biomedical applications across various physiological contexts.

## 2 Results and Discussion

### 2.1 Characterization of SeNPs and Se@PDA NPs

**Figure 1:**
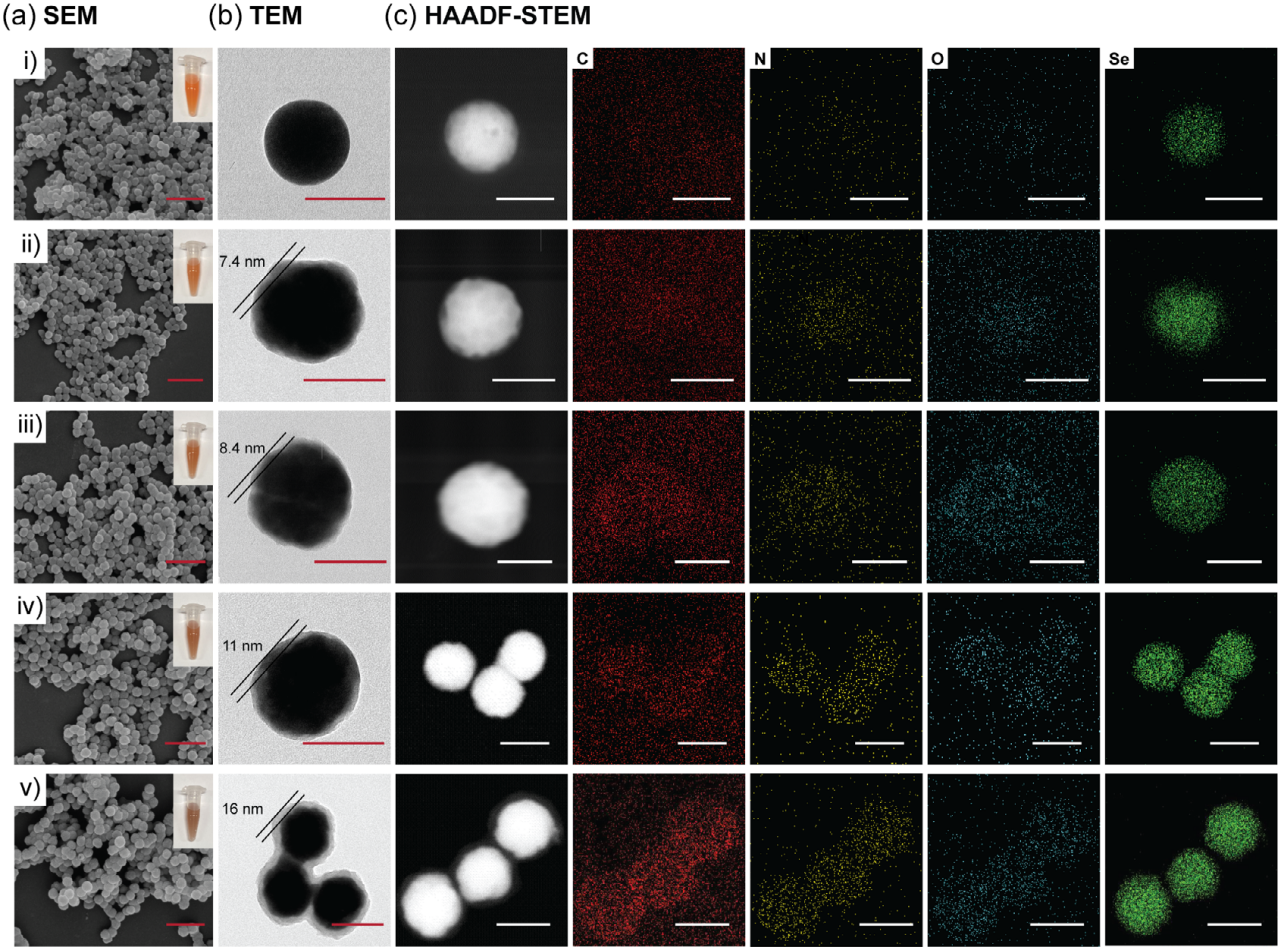
Characterization of i) SeNPs, ii) Se@PDA‐2 NPs, iii) Se@PDA‐4 NPs, iv) Se@PDA‐6 NPs, and v) Se@PDA‐12 NPs including a) SEM (scale bar 500 nm), b) TEM (scale bar 100 nm), and c) HAADF‐STEM with elemental mapping of C, N, O, and Se from left to right (scale bar 100 nm).

SeNPs were synthesized using our previously established method,^[^ ^21^ ^]^ yielding spherical particles with an average diameter of ≈100 nm (**Figure** **1**ai). To synthesize Se@PDA NPs, SeNPs were incubated with dopamine (DA) in Tris‐HCl buffer (pH 8.0), where DA underwent oxidization and polymerization to form PDA. PDA contains catechol and amine functional groups, similar to those used by mussels, which exhibit good adhesion and film‐forming abilities.^[^ ^38^, ^39^ ^]^ Leveraging these adhesive characteristics, PDA self‐assembled on the surface of SeNPs, creating a polymer coating. The coating process was stopped at specific intervals (2, 4, 6, or 12 h) to form PDA coatings of varying thicknesses. These Se@PDA NPs were denoted as Se@PDA‐2 NPs, Se@PDA‐4 NPs, Se@PDA‐6 NPs, and Se@PDA‐12 NPs, respectively. Inset photographs in Figure 1a show a color transition in the Se@PDA NP suspension from orange to dark orange as the coating time increases, indicating the successful polymerization of dopamine. Scanning electron microscopy (SEM) confirmed that the Se@PDA NPs retained their spherical structure after PDA coating (Figure 1a), with the NP diameters increasing as the coating duration extended (Figure S1, Supporting Information). Compared to the uncoated SeNPs, which exhibited a zeta potential of −13.3 mV, the zeta potential initially became less negative (−8.20 mV) after 2 h of PDA coating, then progressively decreased to more negative values of −12.4, −14.7, and −20.9 mV after 4, 6, and 12 h of coating, respectively (Figure S1, Supporting Information). These results indicate the successful coating of PDA onto the SeNP surface. Additionally, the effect of coating time on the PDA thickness was investigated. As shown in Figure 1b, increasing coating time led to a linear increase in the PDA thickness, with the thickness increasing by ≈0.9 nm per additional hour after an initial 2‐h coating period. Finally, to confirm the composition of the coating, elemental mapping of Se@PDA NPs was performed using high‐angle annular dark‐field scanning transmission electron microscope (HAADF‐STEM) (Figure 1c), which showed increased intensities of carbon, oxygen, and nitrogen with longer coating time. Collectively, these characterization results confirm the successful formation and tunable thickness of the PDA coating on SeNPs.

**Figure 2:**
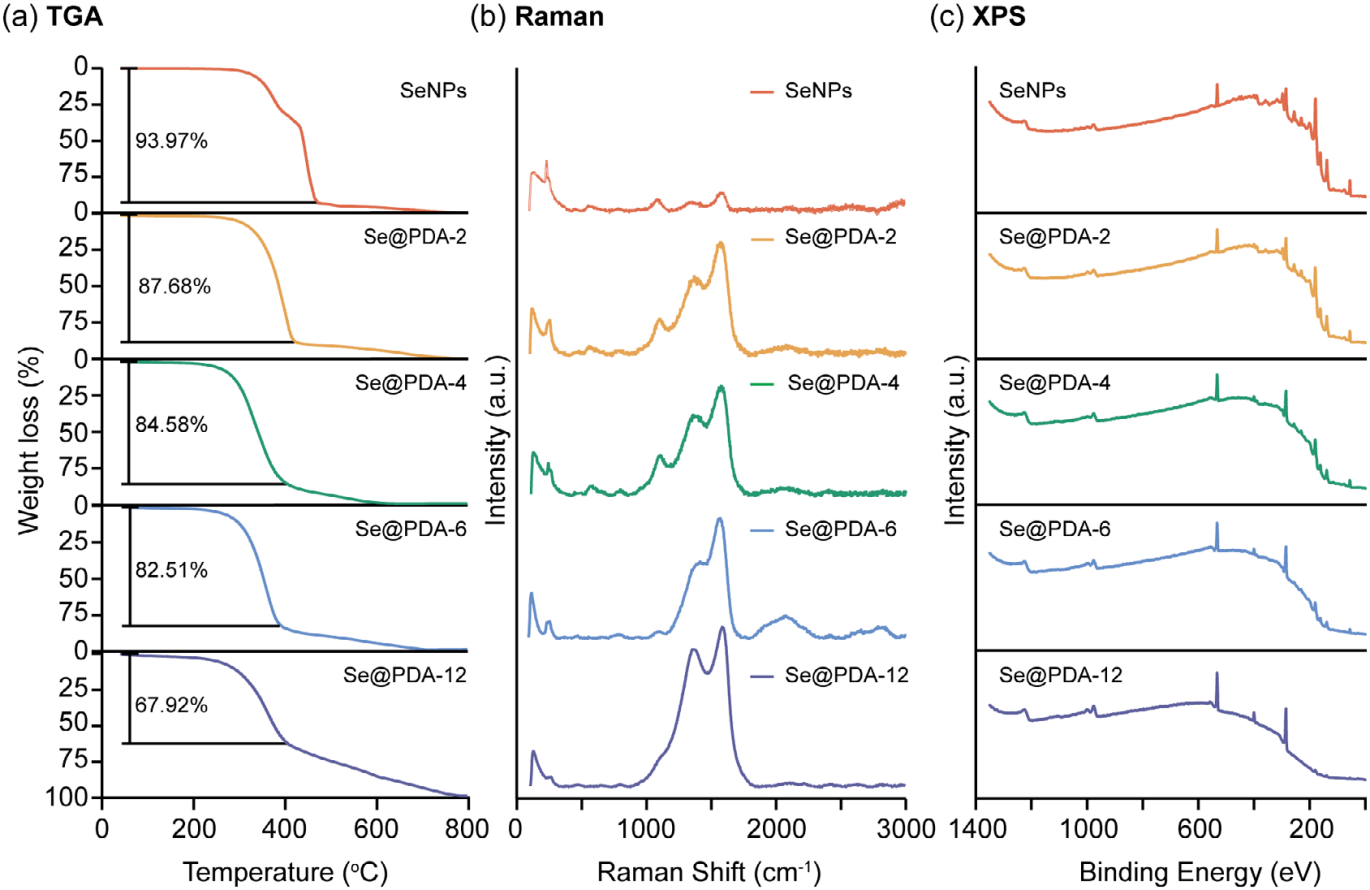
Compositional analysis of SeNPs, Se@PDA‐2 NPs, Se@PDA‐4 NPs, Se@PDA‐6 NPs, and Se@PDA‐12 NPs including a) TGA, b) Raman, and c) XPS.

To further confirm the increased PDA coating thickness with increasing coating time, thermogravimetric analysis (TGA) was carried out (**Figure** **2**a). The initial endothermic stage occurred below 200 °C, with a minor weight loss attributed to water evaporation. SeNPs were almost completely decomposed between 270 and 470 °C, with a weight loss of 93.97%. The corresponding differential thermogravimetry (DTG) curve showed two peaks at 300 and 400 °C, corresponding to the decomposition of PVA^[^ ^40^ ^]^ (the capping agent used in SeNP synthesis) and SeNPs, respectively. In contrast, only partial decomposition of Se@PDA NPs occurred before 400 °C, with weight losses of 87.68%, 84.58%, 82.51%, and 67.92% for Se@PDA‐2 NPs, Se@PDA‐4 NPs, Se@PDA‐6 NPs, and Se@PDA‐12 NPs, respectively. The slow weight loss observed after 400 °C was attributed to the decomposition of PDA. Figure S2a, Supporting Information summarizes the average weight losses of NPs before the breakpoint at 400 °C, reflecting an increasing proportion of PDA in the nanocomposite. Additionally, the DTG curves of SeNPs and Se@PDA NPs (Figure S2b–f, Supporting Information) show decreasing peak values with increasing coating time. This can be attributed to the enhanced physical interaction between SeNPs and the PDA coating, which slowed down the rate of weight loss during heating. Raman spectroscopy was utilized to analyze the molecular structure and composition of SeNPs and Se@PDA NPs (Figure 2b). The peak observed around 230–250 cm^−1^ is attributed to Se‐Se vibration^[^ ^41^ ^]^ and the two distinct peaks around 1390 and 1575 cm^−1^ correspond to the D band and G band, respectively.^[^ ^42^ ^]^ These bands are well‐known features in the Raman spectra of carbon‐based materials. Specifically, the D band represents disorder, while the G band indicates the presence of graphitic structures.^[^ ^43^ ^]^ Due to the heterogeneous polymeric nature of PDA,^[^ ^44^ ^]^ it is challenging to fit the characteristic peaks to each functional group. Therefore, we use the peak intensity ratios at 1575 and 330 cm^−1^ to compare the percentage of organic components in the particles. The increasing I_G_/I_Se_ ratio suggests more PDA coating on the surface of SeNPs (Table S1, Supporting Information).

Additionally, X‐ray photoelectron spectroscopy (XPS) was conducted to analyze the elemental composition of SeNPs and Se@PDA NPs. Figure 2c represents the survey scans of SeNPs, Se@PDA‐2 NPs, Se@PDA‐4 NPs, Se@PDA‐6 NPs, and Se@PDA‐12 NPs. The peaks around 530, 400, 280, and 55 eV correspond to oxygen, nitrogen, carbon, and selenium, respectively. The increase in nitrogen intensity and the decrease in selenium intensity demonstrate the increasing thickness of PDA coating with increasing coating time. This is further supported by the atomic percentages of NPs summarized in Table S2, Supporting Information. High‐resolution region scans of O1s, N1s, C1s, and Se3d provide more details on the chemical states and bonds (Figure S3, Supporting Information). The peak positions, peak intensity ratios, and the corresponding assigned groups are summarized in Table S3, Supporting Information. Additionally, the chemical structures of the building blocks of PDA are illustrated in Figure S4, Supporting Information. The single peak at 532.54 eV in the O1s spectrum of SeNPs is attributed to the C—O—H from PVA, the capping agent used in the synthesis of SeNPs. After PDA coating, a second peak appeared around 531.0 eV, which is fitted to the C=O bond from oxidized dopamine, dopamine‐o‐quinone (DQ).^[^ ^45^ ^]^ As indicated by the percentage of the area under the curve, with increasing coating time, the intensity of the C=O peak increased, which in turn implies more PDA was coated onto the SeNP surface. The three components of the N1s spectra correspond to R‐NH_2_ (amine), R_2_‐NH (pyrrole), and —N= (imine).^[^ ^46^, ^47^ ^]^ No nitrogen was detected from SeNPs. In contrast, after 2 h of coating, nitrogen was detected on the Se@PDA‐2 NP surface in the form of primary amine (401.86 eV) and pyrrole (399.84 eV). The predominate pyrrole suggests the formation of 5,6 dihydroxyindole (DHI) via the cyclized self‐polymerization of dopamine.^[^ ^48^ ^]^ The presence of the primary amine is due to unpolymerized dopamine or the concurrent existence of noncovalently self‐assembled dopamine within the covalently polymerized PDA.^[^ ^49^ ^]^ As the coating time increased, the primary amine was no longer detected, and instead, imine (398.7 eV) was formed. After dopamine was oxidized to DQ, the primary amine group from dopamine or Tris buffer can react with DQ via a Michael‐type addition or Schiff base reaction, forming C=N.^[^ ^50^ ^]^ The imine structure may also be attributed to the tautomeric structure.^[^ ^22^ ^]^ The C1s spectra of particles all showed three peaks, which are fitted into C—C/C=C (284.8 eV), C—O/C—N (286.2 eV), and Se Auger (285.2 eV). Due to the heterogeneous polymeric structure of PDA and overlapping of Se Auger peak with possible C=O peak, the C1s spectra cannot provide more surface characteristics. Lastly, the Se3d spectra were all fitted with one pair of peaks corresponding to the Se3d_3/2_ and Se3d_5/2_ with a separation of 0.86 eV and an intensity ratio of 0.735. This suggests that after coating, the electron state of Se remained at 0, without forming new chemical bonds with other elements. The compositional analysis of SeNPs and Se@PDA NPs demonstrates successful PDA coating, with thickness easily tunable by adjusting the reaction time.

### 2.2 pH‐Dependent NO Generation Using SeNPs

The pH levels in the human body vary depending on the specific region or fluid, maintaining a delicate balance for optimal physiological function. For instance, blood maintains a tightly regulated pH of 7.35–7.45 to ensure proper metabolic processes.^[^ ^51^ ^]^ The stomach's highly acidic pH of 1.5–3.5 aids in digestion,^[^ ^52^ ^]^ while the small intestine's more alkaline pH of 7.4–8.0 facilitates digestive enzyme function.^[^ ^53^ ^]^ In a therapeutic context, conditions such as tumors,^[^ ^54^, ^55^, ^56^ ^]^ inflammation^[^ ^57^ ^]^ or diabetic wounds^[^ ^58^ ^]^ are typically slightly acidic. Moreover, the pH of chronic wounds ranges from 5.45 to 8.65.^[^ ^59^ ^]^ Wound healing involves a complex regenerative process, with the pH environment changing during different phases. In the initial inflammation phase, wounds exhibit low pH, which helps suppress bacterial growth, while during the granulation phase, the pH shifts toward a more basic level. However, in chronic wounds this balance is disrupted, resulting in a persistently high pH.^[^ ^60^ ^]^

**Figure 3:**
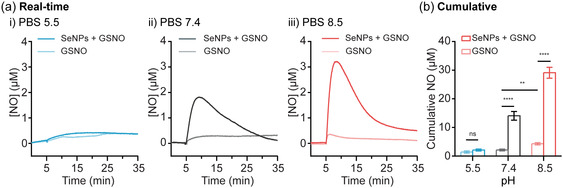
a) Real‐time and b) 4‐h cumulative NO generation from GSNO (50 μM) in PBS buffer (pH 5.5, 7.4, and 8.5) in the presence or absence of SeNPs (40 μg mL^−1^). Statistical significance was calculated using two‐way ANOVA, ns = no significance, **p < 0.01, ****p < 0.0001. *n *= 3; error bars represent standard deviation.

Recognizing that different pH values can significantly alter the catalytic behavior of nanomaterials,^[^ ^61^, ^62^ ^]^ we first investigated the NO‐generating ability of SeNPs in PBS buffer at pH 5.5, 7.4, and 8.5. The pH effect on the NO‐generating ability of Se@PDA NPs will be discussed in Section 2.3. The real‐time NO‐generation profile (**Figure** **3**a–c) reveals a strong dependence on pH, where more NO was generated from GSNO at basic pH levels. Specifically, the peak NO concentration exceeded 3 μM at pH 8.5, whereas at pH 5.5, the NO‐generating ability was significantly inhibited. This trend was also consistent with the 4‐h cumulative NO generation (Figure 3d). At neutral and basic conditions, the cumulative NO generated was significantly higher compared to the control, whereas at acidic pH conditions of 5.5, there was no significant difference. Recent studies investigated the stability of GSNO under different pH conditions, demonstrating that higher pH levels lead to an increased rate of GSNO decomposition, while acidic pH levels enhance GSNO stability.^[^ ^63^, ^64^ ^]^ Figure S5 (Supporting Information) illustrates the electronic structures of two GSNO resonances, the electron‐donating (D) resonance and electron‐withdrawing (I) resonance. Acidic conditions favor the existence of D resonance, which increases the strength of the S—N bond.^[^ ^65^ ^]^ As the pH value increases, the equilibrium shifts to the right, resulting in an increased rate of GSNO decomposition. In addition to the stability of GSNO, the surface chemistry of SeNPs is responsible for this pH‐dependent effect. The SeNPs were synthesized using PVA as a capping agent, where the hydroxyl groups of PVA undergo deprotonation in basic conditions due to OH^−^ ions.^[^ ^66^ ^]^ This deprotonation enhances the nucleophilicity of the SeNP surface, making it more reactive to the S—N bond of GSNO. Conversely, in an acidic environment, the nucleophilic reaction is constrained, leading to reduced reactivity. The effect of pH on the stability of GSNO and the surface chemistry of SeNPs collectively resulted in pH‐dependent NO generation. Specifically, SeNPs exhibit optimal NO generation in basic conditions, whereas their NO‐generating ability was inhibited in acidic conditions.

### 2.3 Tunable NO Generation Using Se@PDA NPs

**Figure 4:**
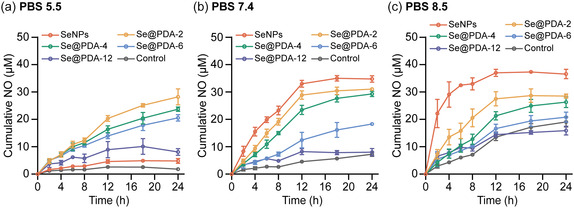
Time‐dependent cumulative NO generation from GSNO (50 μM) catalyzed by 40 μg mL^−1^ of SeNPs or Se@PDA NPs in PBS at pH a) 5.5, b) 7.4, and c) 8.5. *n *= 3; error bars represent standard deviation.

The pH‐responsive behavior of SeNPs enables selective and effective NO delivery under neutral and basic conditions. However, it is crucial to develop a NO delivery platform that is versatile enough to be applicable across a wider pH range. This versatility would facilitate therapeutic applications such as anticancer, antibacterial, anti‐inflammation, and wound healing treatments, where the pH range may vary from acidic to basic. To address this challenge, we employed a coating of PDA with varying thicknesses on SeNPs. **Figure** **4** illustrates the cumulative NO generation catalyzed by SeNPs or Se@PDA NPs in PBS at pH 5.5, 7.4, and 8.5. The NO‐generation kinetic decreased with increasing thickness of the PDA coating, as the PDA shell acted as a barrier between the active catalytic site and GSNO. This also demonstrates that the NO‐generating rate can be tuned by using Se@PDA NPs with varying PDA thickness, making them suitable for biomedical applications where the amount of NO needs to be precisely controlled. Additionally, the PDA coating altered the surface chemistry of SeNPs, thus affecting their NO‐generating capacity. Specifically, at pH 5.5, Se@PDA‐2 NPs exhibited significantly higher NO‐generating activity compared to SeNPs (Figure 4a). This can be attributed to the increased reactivity of Se@PDA NPs resulting from the presence of phenolic hydroxyl and amine groups in the PDA,^[^ ^67^ ^]^ which leads to an increased affinity toward GSNO, thereby promoting its decomposition. A similar study involving PDA‐modified carbon nanotubes also demonstrated that the PDA coating improved catalytic activity in ethylbenzene dehydration. This enhancement was due to the electron transfer from PDA to the nanocarbon matrix, which increased the nucleophilicity of oxygen functionalities on the nanocarbon matrix.^[^ ^68^ ^]^ As discussed in Section 2.2, the NO generating capacity of SeNPs is pH‐dependent, exhibiting enhanced NO‐generating capacity in basic conditions. However, coating SeNPs with PDA mitigated this pH‐dependent effect, leading to a consistent NO generation across a pH range of 5.5–8.5 (Figure 4b–c). It is worth noting that NO generation by SeNPs at pH 8.5 was faster. Specifically, 22.13 μM of NO was generated within 2 h, compared to 8.37 μM at pH 7.4. Although NO is a therapeutic gas, high NO levels can lead to oxidative stress, cell apoptosis,^[^ ^69^ ^]^ and DNA damage.^[^ ^70^ ^]^ The PDA coating is therefore shown to regulate the NO generation rate and avoid a burst generation. Overall, the PDA coating successfully mitigated the pH‐dependent limitation on the NO‐generating ability of SeNPs, enhancing NO‐generation at pH 5.5 and preventing burst NO generation at pH 8.5, thereby ensuring efficacy in both acidic and basic environments.

### 2.4 Stability in Physiological Conditions

**Figure 5:**
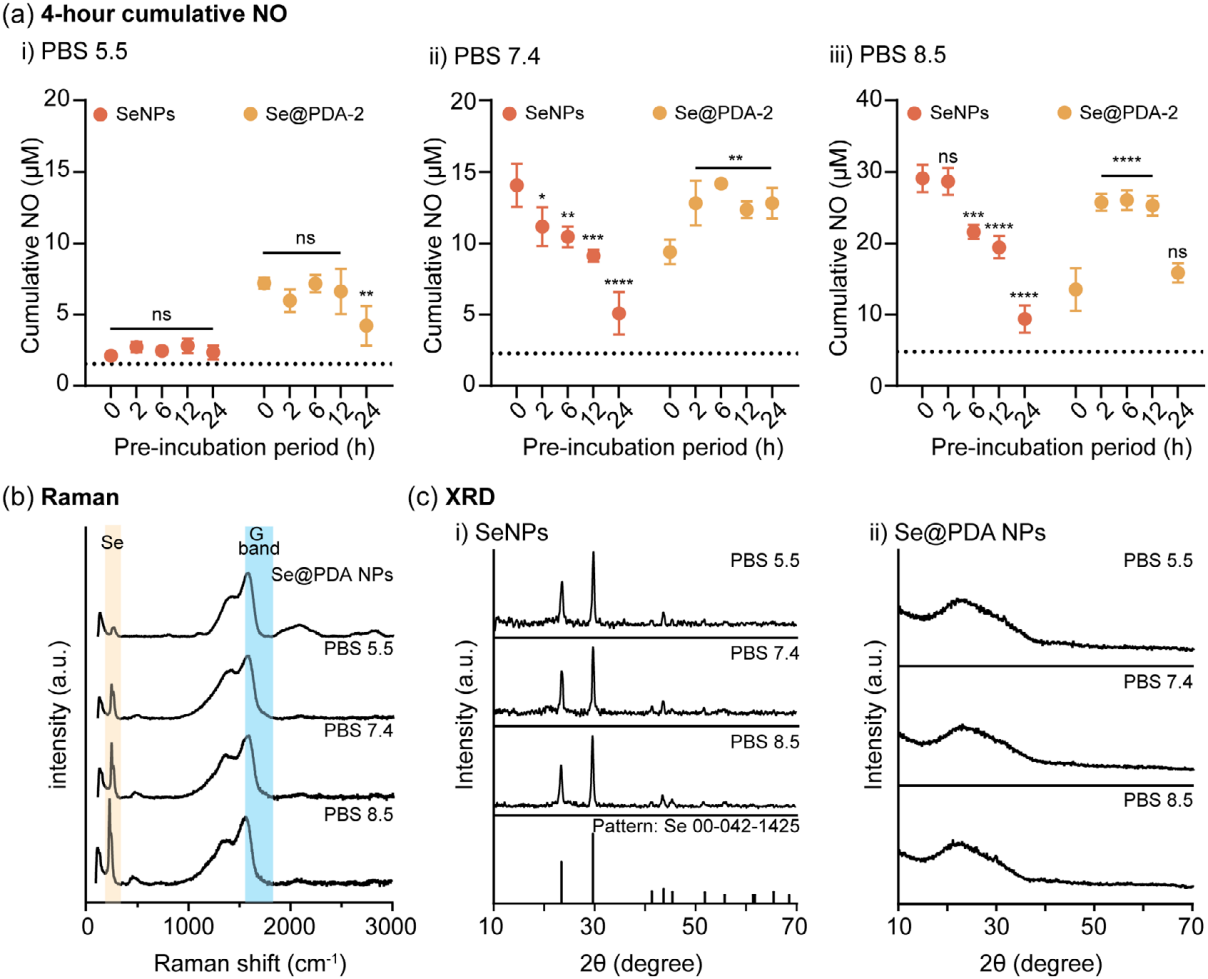
a) Four‐hours cumulative NO generation from GSNO (50 μM) catalyzed by 40 μg mL^−1^ of SeNPs or Se@PDA‐2 NPs after incubating in PBS buffer at pH i) 5.5, ii) 7.4, and iii) 8.5, for 0, 2, 6, 12, and 24 h. The dashed line represents the control (GSNO) in corresponding conditions. b) Raman spectra of Se@PDA‐6 NPs before and after 12 h of pre‐incubation in PBS at pH 5.5, 7.4, and 8.5. c) XRD of i) SeNPs and ii) Se@PDA‐2 NPs after 6 h of incubation with GSNO (50 μM) in PBS buffer at pH 5.5, 7.4, and 8.5. Statistical significance was calculated using one‐way ANOVA, ns = no significance, *p < 0.05, **p < 0.01, ***p < 0.001, ****p < 0.0001. *n *= 3; error bars represent standard deviation.

In our previous study, we identified a limitation regarding the decrease in NO‐generating activity of SeNPs after extended incubation in physiologically relevant conditions (PBS). This decline was attributed to particle aggregation and the transition of particle structure from amorphous to crystalline. To address this issue, the PDA coating was evaluated for its ability to retain the NO‐generating properties of Se@PDA NPs, even after long incubation periods, by enhancing the nanomaterial's stability.^[^ ^71^, ^72^, ^73^ ^]^ Specifically, SeNPs or Se@PDA NPs were incubated in PBS buffer at varying pH levels for different durations. Following the incubation, GSNO was introduced, and the 4‐h cumulative NO generation was measured to assess their catalytic activity. **Figure** **5**a illustrates the 4‐h cumulative NO generation catalyzed by SeNPs or Se@PDA‐2 NPs after incubation in PBS pH 5.5, 7.4, and 8.5 for 0, 2, 6, 12, and 24 h. Consistent with previous findings, at pH 5.5 Se@PDA‐2 NPs demonstrated enhanced NO‐generating ability compared to SeNPs. After up to 12 h of pre‐incubation in PBS buffer, Se@PDA‐2 NPs showed no significant decrease in NO‐generating ability, indicating their improved stability at pH 5.5. At pH 7.4 and 8.5, NO generation catalyzed by SeNPs decreased after pre‐incubation, whereas Se@PDA‐2 NPs maintained their NO‐generating ability even after 24 h of pre‐incubation in PBS. Additionally, a significant increase in NO generation was observed after 2, 6, and 12 h of pre‐incubation. The cumulative NO generation catalyzed by Se@PDA‐4 NPs, Se@PDA‐6 NPs, and Se@PDA‐12 NPs after pre‐incubation in PBS buffer at different pH levels is presented in Figure S6, Supporting Information. Se@PDA‐12 NPs exhibited the lowest activity due to their thicker PDA coating, which further hindered the interaction between the active Se sites and GSNO. However, they were stable in the PBS buffer, showing no significant decrease in NO generation after up to 12 h of pre‐incubation. At pH 5.5 and 7.4, Se@PDA‐4 and Se@PDA‐6 demonstrated no decrease in catalytic activity after up to 12 h of pre‐incubation. At pH 8.5, they exhibited enhanced activity following pre‐incubation, likely due to the exposure of more active catalytic sites, like what was observed with Se@PDA‐2. Overall, Se@PDA NPs exhibited greater stability in PBS buffer across a pH range of 5.5–8.5 compared to SeNPs, demonstrating their potential for use under physiological conditions.

To investigate potential changes in surface properties, Raman spectroscopy was carried out on Se@PDA‐6 NPs after pre‐incubation in PBS at pH 5.5, 7.4, and 8.5 (Figure 5b). The peak positions and intensity ratios of the G band and Se are summarized in Table S4, Supporting Information. The peak intensities corresponding to Se increased after pre‐incubation, and the I_G_/I_Se_ ratio of Se@PDA‐6 NPs decreased from 7.50 to 1.82, 1.12, and 0.78 after 12 h of pre‐incubation in PBS at pH 5.5, 7.4, and 8.5, respectively, indicating that more active sites were exposed. To further characterize the crystal structure of NPs after the reaction, XRD was carried out to examine any changes in the crystal structure. Nanoparticles after the reaction were centrifuged and redispersed in ultrapure water to remove any salt that can interfere with the XRD analysis. Before incubation, XRD showed that all the particles were in an amorphous structure, as indicated by the broad peak from 20° to 30° (Figure S7, Supporting Information). Following a 6‐h incubation in PBS with GSNO, consistent with our previous study, SeNPs underwent a shift to crystalline structure, evident from the appearance of sharp peaks in XRD spectra (Figure 5ci). The characteristic peaks are indexed to the pattern (Se 00‐042‐1425). Conversely, Se@PDA‐2 NPs remained in an amorphous structure after incubating under different pH conditions (Figure 5cii). These observations indicate that the PDA shell acts as a barrier, preventing structural changes and thereby retaining their superior catalytic activity.

### 2.5 Biocompatibility and Cellular Uptake

**Figure 6:**
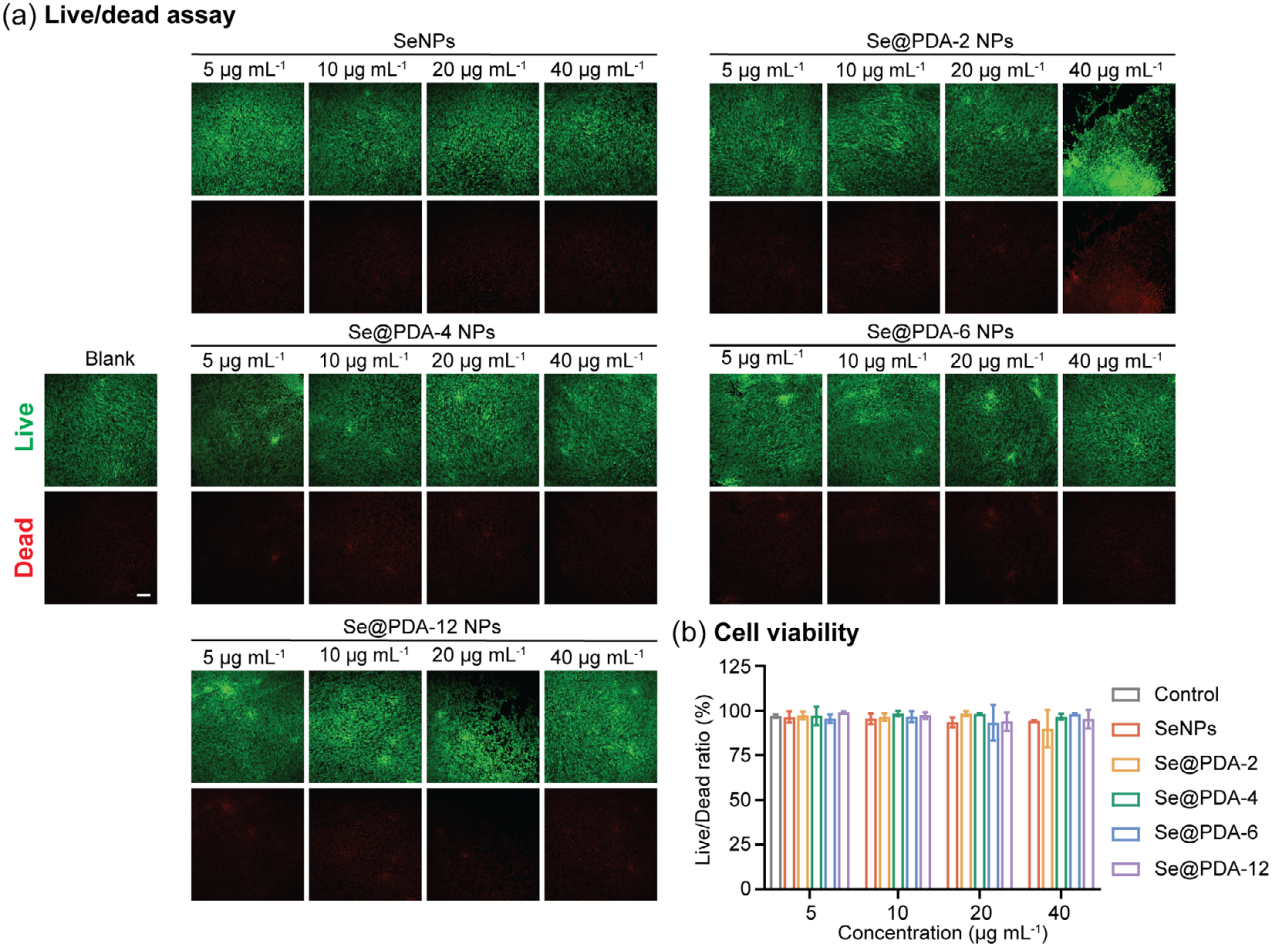
a) Fluorescence microscope images of NIH 3T3 cells after 48 h of incubation with SeNPs, Se@PDA‐2 NPs, Se@PDA‐4 NPs, Se@PDA‐6 NPs, or Se@PDA‐12 NPs at various concentration (5, 10, 20, and 40 μg mL^−1^). Cells stained with calcein (green) indicates live cells and ethidium (red) indicates dead cells. (Scale bar 150 μm). b) Corresponding cell viability analysis from Live/Dead assay. *n *= 5; error bars represent standard deviation.

Se@PDA NPs with enhanced stability show potential as an injectable drug for NO delivery. Their suitability was initially assessed through a biocompatibility test, followed by evaluations of cellular uptake and intracellular NO level to confirm their NO‐generating ability *in vitro*. The biocompatibility of the SeNPs and Se@PDA NPs was examined by evaluating their cytotoxicity toward mouse embryonic fibroblast NIH 3T3 cells, which is often used for preliminary screening of materials prior to *in vivo* evaluation.^[^ ^74^ ^]^ The Live/Dead assay was employed to evaluate cell viability (**Figure** **6**a). No decrease in cell viability was observed after 48 h of incubation with SeNPs at concentrations ranging from 5 to 40 μg mL^−1^, demonstrating their high biocompatibility. Noticeably, when cultured with Se@PDA‐2 NPs at 40 μg mL^−1^, cell aggregation was observed in some samples along with an increase in the number of dead cells, suggesting interactions between the nanoparticles and cells that triggered various cellular responses. Therefore 20 μg mL^−1^ was selected as the highest concentration for the following *in vitro* test. However, the quantitative analysis of the Live/Dead assay (Figure 6b) revealed that the average cell viability of Se@PDA‐2 NPs at 40 μg mL^−1^ remained above 90%, demonstrating that while Se@PDA NPs may trigger some cellular responses at high concentrations, it remained biocompatible. Further cytotoxicity evaluation using AlamarBlue confirmed that both SeNPs and Se@PDA NPs were biocompatible at concentrations from 5 to 40 μg mL^−^^1^, with cell viability exceeding 95% (Figure S8, Supporting Information). These findings demonstrate that SeNPs and Se@PDA NPs are suitable for biomedical applications due to their high biocompatibility and minimal cytotoxic effects.

**Figure 7:**
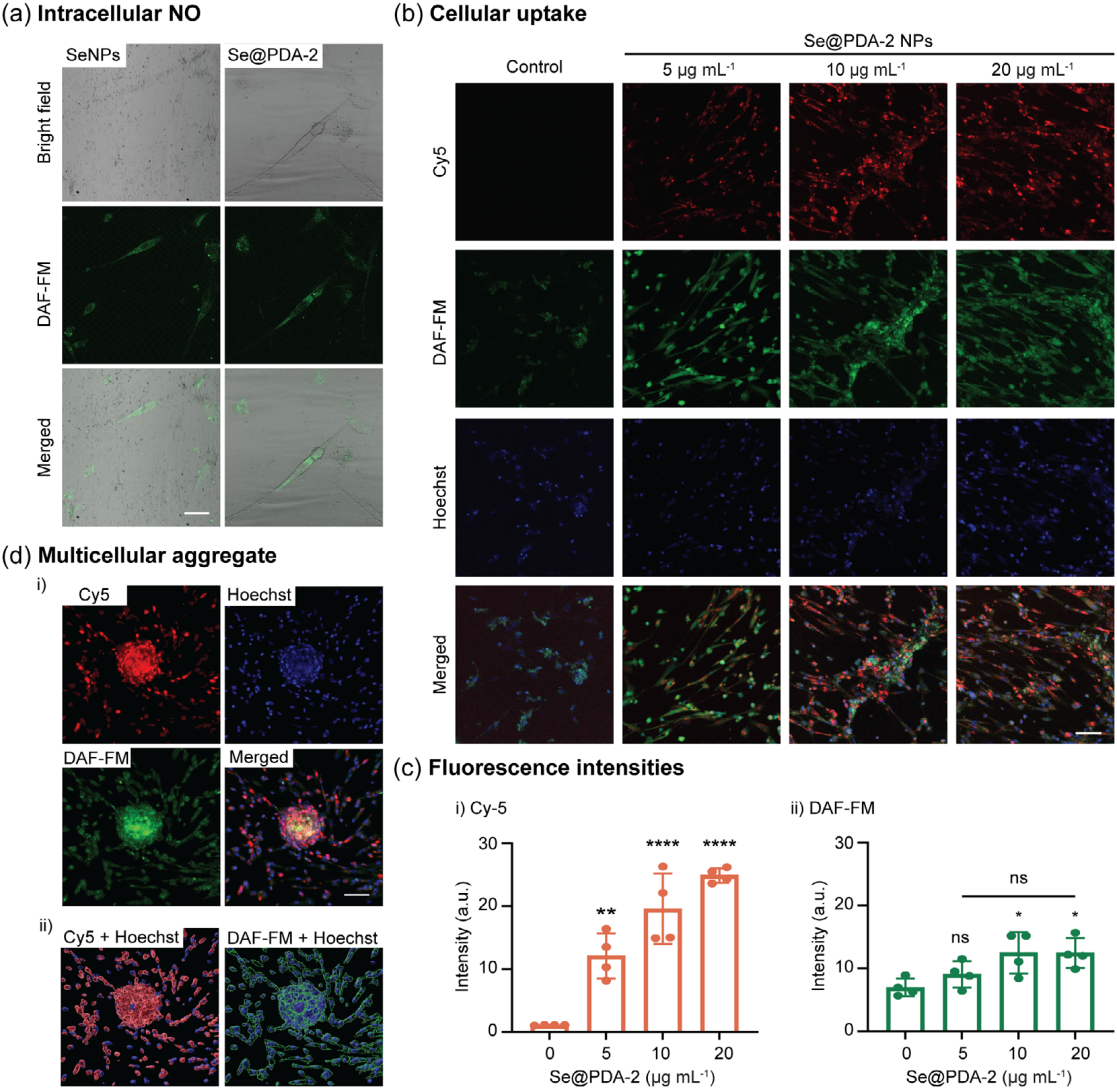
Evaluation of intracellular NO generation, cellular uptake, and multicellular aggregate formation in HCASMCs treated with SeNPs and Se@PDA‐2 NPs. a) Confocal microscope images of HCASMCs incubated with SeNPs or Se@PDA‐2 NPs at 20 μg mL^−1^ stained with DAF‐FM (green) for intracellular NO detection. b) Cy5‐labeled (red) Se@PDA‐2 NPs (5, 10, and 20 μg mL^−1^) stained with DAF‐FM (green) for NO detection and Hoechst (blue) for cell nuclei. c) Corresponding fluorescence intensity (a.u.) analysis of i) Cy5 and ii) DAF‐FM. d) Formation of multicellular aggregates in HCASMCs treated with Cy5‐labeled Se@PDA‐2 NPs (20 μg mL^−1^), i) confocal microscope images and ii) surface co‐localization simulation from Imaris. (Scale bar 100 μm). Statistical significance was calculated using one‐way ANOVA, ns = no significance, *p < 0.05, **p < 0.01, ****p < 0.0001. *n* = 4; error bars represent standard deviation.

After demonstrating the enhanced stability and high biocompatibility of Se@PDA NPs, we further investigated their *in vitro* NO‐generating capacity at the cellular level through incubation with HCASMCs without the addition of GSNO. This setup indicated NO generation by the NPs occurred exclusively from endogenous NO donors at the biorelevant level. Se@PDA‐2 NPs were selected for comparison with SeNPs, due to their high NO‐generating capacity and the ability to induce noticeable cell aggregation. After a 48‐h incubation, the cells were incubated with DAF‐FM, a NO‐sensitive fluorescent dye. The confocal images revealed that both SeNPs and Se@PDA‐2 NPs (20 μg mL^−1^) can induce intracellular NO generation, with SeNPs dispersed in the cell medium and Se@PDA‐2 NPs primarily attached to the cell membrane (**Figure** **7**a). This observation aligns with findings from a recent study investigating the binding between cells and PDA‐coated NPs.^[^ ^32^ ^]^ The catechol and amine groups in the PDA collectively enhance the binding of NPs to dopamine receptors, particularly D2 (DRD2), which in turn promotes cellular uptake. To further visualize the interactions between NPs and the cells and evaluate cellular uptake, Cy5‐labeled Se@PDA‐2 NPs at concentrations of 5, 10, and 20 μg mL^−1^ were incubated with HCASMCs for 48 h. Confocal microscope images revealed a higher Cy5 intensity with increasing concentrations of Cy5‐labeled Se@PDA‐2 NPs (Figure 7b), demonstrating concentration‐dependent cellular uptake (Figure 7ci). The intracellular NO levels measured by DAF‐FM were elevated for Se@PDA‐2 NP groups compared to the control group (Figure 7b,cii). While Se@PDA‐2 NPs at 10 and 20 μg mL^−1^ were both significantly higher than the control group, there was no significant difference between these two concentrations (Figure 7cii). This implies that Se@PDA‐2 NPs‐induced intracellular NO levels but did not exhibit a concentration‐dependent trend, likely due to the limited availability of NO donors within the cellular environment.^[^ ^7^ ^]^ Given that excessive NO levels can induce cellular stress and cell apoptosis,^[^ ^75^ ^]^ and considering the high biocompatibility of Se@PDA‐2 NPs demonstrated in Figure 6, these results suggest that Se@PDA‐2 NPs can significantly induce intracellular NO at physiologically relevant levels, thereby maintaining cell viability. The formation of multicellular aggregates was observed after treatment with 20 μg mL^−1^ of Se@PDA‐2 NPs (Figure 7di), suggesting enhanced cell interactions and the establishment of cell communication networks. The surface co‐localization simulation further demonstrated the cellular uptake of Se@PDA‐2 NPs and induced NO generation in both the nucleus and the cytoplasm (Figure 7dii). The immunostaining results indicated enhanced cellular uptake of Se@PDA‐2 NPs, their ability to generate NO within cells, and the formation of multicellular aggregates, which could have implications for cell phenotypic switches for tissue engineering and therapeutic applications.

### 2.6 Sustained NO Generation and Long‐Term Stability

**Figure 8:**
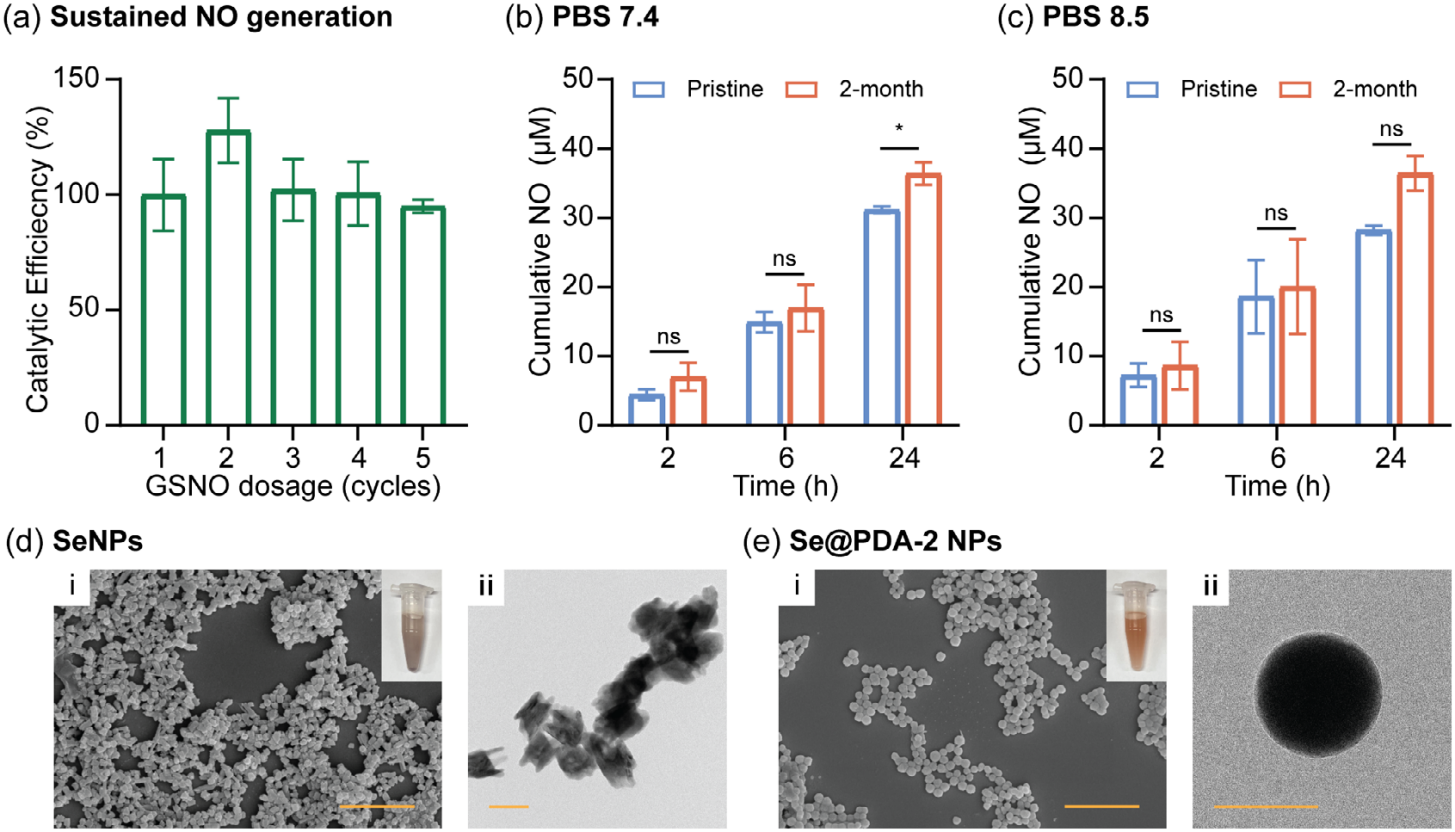
a) Catalytic efficiency of Se@PDA‐2 NPs upon five additions of GSNO (12.5 μM) at 37 °C in PBS pH 7.4. Cumulative NO generation from GSNO (50 μM) catalyzed by pristine and 2‐month stored Se@PDA‐2 NPs (40 μg mL^−1^) in b) PBS pH 7.4 and c) PBS pH 8.5. i) SEM and ii) TEM images of 2‐month stored d) SeNPs and e) Se@PDA‐2 NPs after 12 h of incubation with GSNO (50 μM) and GSH (1 mM) in PBS pH 7.4 (SEM scale bar, 1 μm; TEM scale bar, 100 nm). Statistical significance was calculated using two‐way ANOVA, ns = no significance, *p < 0.05. *n *= 3; error bars represent standard deviation.

Sustained NO generation is a crucial feature affecting the practical use of Se@PDA NPs, particularly when applied as injectable drugs in the biomedical field where persistent NO is required. To investigate this property, an electrochemical NO probe was used to measure the real‐time NO generation upon five successive doses of GSNO. Since Se@PDA‐2 NPs demonstrated enhanced cellular uptake and higher NO‐generating ability compared with other Se@PDA NPs, we focused solely on measuring the sustained performance of Se@PDA‐2 NPs. The NO generation rate of Se@PDA‐2 NPs was lower than that of SeNPs, requiring ≈6 h for the current to return to baseline, with NO still being generated after 3 h (Figure S9a, Supporting Information). Hence, adding five doses of GSNO after the current fully returned to baseline would take more than 15 h. Considering the limitation of monitoring NO probes over long periods, we added GSNO (12.5 μM) approximately every 1.5 h. As illustrated in Figure S9b (Supporting Information), the peak rise after each dosage of GSNO was comparable, indicating sustained NO generation. The average peak rises of each GSNO dosage from three repeats were calculated and compared with the first dosage as a percentage (**Figure** **8**a), showing that the catalytic efficiency of Se@PDA‐2 NPs did not decrease during these five reaction cycles. Furthermore, since glutathione (GSH) can lead to the degradation of PDA,^[^ ^76^, ^77^ ^]^ we also investigated the sustained NO generation in the presence of GSH. As shown in Figure S10 (Supporting Information), both the real‐time NO generation profile and catalytic efficiency demonstrated that Se@PDA‐2 NPs exhibited sustained NO‐generating capacity in the presence of GSH at bio‐relevant concentrations (1 mM).

Finally, the long‐term stability of the particles was investigated after storing them in ultrapure water at 4 °C for 2 months. The NO‐generating capacities were compared with the pristine particles. As depicted in Figure 8b–c, the 2‐month stored Se@PDA‐2 NPs showed no significant decrease in cumulative NO generation within 2, 6, and 24 h in PBS at pH 7.4 and 8.5, respectively. SeNPs, Se@PDA‐4 NPs, Se@PDA‐6 NPs, and Se@PDA‐12 NPs were also stable for at least 2 months, showing no significant decrease in NO‐generating activity (Figure S11, Supporting Information). Moreover, XRD was performed to investigate any structural changes after long‐term storage. As shown in the XRD spectra (Figure S12, Supporting Information), the particles retained their amorphous structure before and after incubation with GSNO. SEM and TEM further illustrated the morphology of SeNPs and Se@PDA NPs after incubation with GSNO. SeNPs shifted from a regular spherical shape to an irregular shape (Figure 8d), while Se@PDA NPs maintained their spherical structures and were less aggregated (Figure 8e and Figure S13, Supporting Information). Additionally, the inset photographs suggest that SeNPs were aggregated after incubation, leading to a color shift from orange to dark gray. Overall, the characterizations suggest that the PDA coating effectively prevents aggregation and promotes long‐term stability.

## 3 Conclusions

Catalytic NO generation from endogenous NO donors using nanomaterials has emerged as a highly effective strategy for spatiotemporal NO delivery. Developing nanomaterials that are stable in physiological conditions is crucial for sustained NO generation. This study demonstrated that coating SeNPs with PDA mitigated pH dependency, enabling NO generation over a broad pH range (5.5–8.5) and preventing burst NO generation. Se@PDA NPs exhibited improved stability in PBS buffer, offering a stable and effective platform for therapeutic applications. The biocompatibility of Se@PDA NPs was investigated against NIH 3T3 cells, showing high cell viability as measured by both Live/Dead and AlamarBlue assays. Notably, interactions between the PDA coating and cellular dopamine DRD2 receptors promoted binding and uptake, leading to increased intracellular NO levels in HCASMCs. The formation of multicellular aggregates was observed after treatment with Se@PDA NPs, suggesting the establishment of cell communication networks. Additionally, the sustained NO generation and long‐term stability of Se@PDA NPs were evaluated, showing no decrease in NO‐generating ability over time. This study overcomes the limitations of SeNPs and presents the potential of Se@PDA NPs in various biomedical applications where pH variability is a factor, such as wound areas, tumor tissues, and inflammatory environments. Future research could explore the intracellular effects and signaling pathways of the catalytic generation of NO after Se@PDA cellular uptake, promoting clinical applications of this promising nanotechnology.

## 4 Experimental Section

4.1

4.1.1

##### Materials

Sodium selenite (Na_2_SeO_3_), ascorbic acid (C_6_H_8_O_6_), polyvinyl alcohol (PVA; Mw 90–98 kDa), dopamine hydrochloride, glutathione reduced (GSH), triethylamine, Griess reagent (modified), phosphate‐buffered saline (PBS), and Tris hydrochloride (Tris‐HCl) were purchased from Sigma‐Aldrich. *S*‐Nitrosoglutathione (GSNO) was purchased from MedChemExpress. 1‐Ethyl‐3‐(3‐dimethylaminopropyl)carbodiimide hydrochloride (EDC‐HCl) was purchased from Thermo Fisher Scientific. Ultrapure water (18.2 MΩ cm resistance) provided by arium mini Sartorius was used throughout the experiments. All reagents were used without further purification.

##### Synthesis of SeNPs and Se@PDA NPs

SeNPs were synthesized as per our previous protocol.^[^ ^21^ ^]^ Briefly, 5 mL of sodium selenite (100 mM) and 5 mL of PVA (1 mg mL^−1^) were mixed at 800 RPM at room temperature. Then, 10 mL of ascorbic acid (250 mM) was introduced into the above mixture at a flow rate of 4 mL min^−1^, and the solution was stirred for another 30 min under the same conditions. The resulting SeNPs were collected by centrifugation at 9500 RPM for 15 min and washed three times with ultrapure water. To synthesize Se@PDA NPs, 1.6 mL of SeNPs (2.5 mg mL^−1^) were mixed with 13.8 mL Tris‐HCl (0.1 M, pH 8.0) and 4 mL ethanol. After 5 min stirring at 21 °C and 400 RPM, 0.6 mL of dopamine‐HCl (10 mg mL^−1^) was added. The mixture was stirred for 2, 4, 6, or 12 h at 1000 RPM to form PDA coatings of various thicknesses. The polymerization and coating process was stopped by washing with ultrapure water three times via centrifugation at 8000 RPM for 15 min. The Se@PDA NPs were denoted as Se@PDA‐2, Se@PDA‐4, Se@PDA‐6, and Se@PDA‐12 and stored at 4 °C for future use.

##### Preparation of Cy5‐Labeled Se@PDA‐2 NPs

To prepare Cy5‐labeled Se@PDA‐2 NPs, 1.8 mL of Se@PDA‐2 NPs (1 mg mL^−1^) was incubated with 0.02 mL of Cy5‐COOH (10 mg mL^−1^), 0.1 mL of EDC‐HCl (10 mg mL^−1^), 0.02 mL of triethylamine, and 0.06 mL of ultrapure water. This mixture was kept in an ice bath, protected from light, and stirred at 600 RPM for 24 h. Following the reaction, the mixture was dialyzed against ultrapure water using tubing with MWCO 1 kDa for 48 h to remove unreacted reagents, with the ultrapure water replaced every 12 h.

##### Characterization of Se@PDA NPs

The morphology of Se@PDA NPs was characterized using field emission scanning electron microscopy (FESEM, FEI Nova NanoSEM 230) with an acceleration voltage of 10 kV and a spot size of 3. Sample preparation was carried out by pipetting 10 μL of the NP solution (2 mg mL^−1^ in ultrapure water) onto silica wafers and drying overnight at room temperature. After drying, all samples were coated with a 15 nm platinum layer using a Leica ACE600 sputter coater. The size distribution of SeNPs was determined from SEM images and analyzed using Gaussian fitting. The PDA coating was characterized using transmission electron microscopy (JEOL F200 TEM) with an accelerating beam voltage of 200 kV. The compositions of SeNPs and Se@PDA NPs were characterized by elemental mapping using TEM operating in high‐angle annular dark‐field scanning transmission electron microscope (HAADF‐STEM) mode. The zeta potential of SeNPs and Se@PDA NPs was determined using a Malvern Zetasizer Nano. Nanoparticles were dispersed in ultrapure water (pH 5.14) at a concentration of 50 μg mL^−1^, and measurements were carried out at 25 °C.

Thermogravimetric analysis (TGA) was conducted using a TGA Q5000. Each sample (2.0 mg) was heated to 800 °C with a heating rate of 10 °C min^−1^ under nitrogen purging (25 mL min^−1^). Raman spectra were collected via a Raman spectrometer (inVia Raman microscope, Renishaw) utilizing a 532 nm laser source, 1% laser power, and 1800 gr mm^−1^ grating. For each sample, the acquisition time was 30 s. The composition of Se@PDA NPs was analyzed by X‐ray photoelectron spectroscopy (XPS, Thermo ESCALAB250Xi) with a monochromated Al Kα X‐ray radiation source (energy 1486.68 eV) operating at 120 W. The pass energy for survey scans and region scans were 100 and 20 eV, respectively. X‐ray diffraction (XRD) analysis was carried out with an Empyrean 1 (PANalytical) Thin‐Film XRD at 40 kV and 40 mA with a Cu‐Kα radiation source.

##### NO Generation Using SeNPs and Se@PDA NPs

The Griess assay was used to measure cumulative NO generation. In this assay, suspensions of SeNPs or Se@PDA NPs (40 μg mL^−1^) were mixed with GSNO (50 μM) in 1 mL of PBS buffer (10 mM, pH 7.4). The mixture was incubated at 37 °C protected from light for various time intervals, followed by centrifugation at 9000 RPM for 5 min. Then, 75 μL of the supernatant was mixed with 75 μL of Griess reagent (40 mg mL^−1^ in PBS) and incubated for 15 min protected from light. The absorbance was measured at 546 nm using a SpectraMax M5 microplate reader. The concentration of NO was determined using a calibration curve.

For continuous monitoring of NO generation in real‐time, a free radical analyser (TBR4100, World Precision Instruments) equipped with a NO‐sensitive probe (ISO‐NOP, World Precision Instruments) was used. Changes in current were recorded and converted into NO concentrations using a calibration curve. To obtain the profiles of NO generation, the NO electrode was first immersed in a 3.95 mL particle suspension in PBS (10 mM, pH 7.4) at 37 °C until a stable baseline was established (30–45 min). Then, 50 μL of 1 mM GSNO was added to the suspension to reach a final concentration of 12.5 μM. The changes in current were monitored over time, with the entire reaction conducted at 37 °C, protected from light, and continuously stirred at 400 RPM.

##### Biocompatibility of Se@PDA NPs

The biocompatibility of SeNPs and Se@PDA NPs was investigated using NIH 3T3 cells (below passages 20) from American Type Culture Collection (ATCC). Live/Dead assays (Invitrogen) and AlamarBlue assays (Thermo Fisher) were performed to assess biocompatibility. NIH 3T3 cells were seeded in Dulbecco's Modified Eagle Medium (DMEM, Life Technologies), supplemented with 10% v/v bovine calf serum (BCS, Sigma Aldrich), 0.25 mg mL^−1^L‐Glutamine (Sigma Aldrich), and 1% v/v penicillin/streptomycin (Pen‐Strep, Sigma Aldrich). Cells were sub‐cultured at 80–90% confluency. The NIH 3T3 cells were seeded at a density of 5 × 10^3^ cells 100 μL^−1^ well^−1^ in 96‐well plates and incubated overnight at 37 °C in a 5% CO_2_ incubator for 24 h. Then the cell culture medium was replaced with fresh cell medium containing NPs at concentrations ranging from 5 to 40 μg mL^−1^. Note, the stock solutions SeNPs and Se@PDA NPs (2 mg mL^−1^) were sterilized under a UV lamp for 30 min prior to use. After a 48‐h incubation, cell supernatants were removed, and cells were washed with PBS once to eliminate residual NPs. Then a Live/Dead assay or AlamarBlue assay was carried out.

For the Live/Dead assay, cell medium containing calcein AM (2 μM) and ethidium homodimer‐1 (4 μM) was added to each sample and incubated at 37 °C in a 5% CO_2_ incubator for 45 min. Then the samples were washed with PBS once and imaged using a Zeiss LSM 800 confocal microscope, and the numbers of live or dead cells were counted using Imaris (Oxford Instruments) software. Cell viability was calculated based on the following equation

(1) $$\text{Cell viability }\%=\frac{\text{Live cells}}{\text{Live cells}+\text{Dead cells}}\times 100\%$$

For the AlamarBlue assay, 100 μL of fresh cell medium containing 10% AlamarBlue was added to each well, which was then incubated at 37 °C in a 5% CO_2_ incubator for 3 h. The fluorescence values (Ex/Em = 560/590 nm) were measured using a SpectraMax M5 microplate reader and the cell viability was calculated based on the following equation:

(2) $$\text{Cell viability }\%=\frac{I_{s}}{I_{c}}\times 100\%$$

where *I*_s_ represents the fluorescence of the experimental group and *I*_c_ represents the fluorescence of control groups.

##### Cellular Uptake of Se@PDA NPs and Endogenous NO Generation

The cellular uptake of NPs and *in vitro* NO generation was evaluated using human coronary artery smooth muscle cells (HCASMCs, 350–05A, Cell Applications). Cells were maintained in human SMC growth medium (311–500, Cell Applications) and passaged at 70–90% confluency. HCASMCs were seeded at 2 × 10^4^ cells well^−1^ in 24‐well tissue culture plates for 48 h prior to incubation with Cy5‐labeled Se@PDA‐2 NPs at concentrations ranging from 5 to 20 μg mL^−1^ in fresh medium. After 48 h, the cells were stained with nuclear counterstain Hoechst 33342 (62249, Thermo Fisher, 1 : 500 dilution) and NO fluorescent probe DAF‐FM diacetate (D23842, Thermo Fisher, 5 μM). Imaging was conducted, and the fluorescence intensity and localization were processed using Imaris software.

##### Stability of Se@PDA NPs

To investigate the stability of SeNPs and Se@PDA NPs in physiologically relevant conditions (PBS), the NPs were incubated in PBS at different pH levels for various time intervals. Following incubation, GSNO was added to the particle suspension (1 mL) at a final concentration of 50 μM and further incubated for 4 h. The cumulative NO generation over this 4‐h period was then determined using the Griess assay. The long‐term stability of SeNPs and Se@PDA NPs was evaluated after storing them in ultrapure water at 4 °C for 2 months. Following storage, the NPs at 40 μg mL^−1^ were incubated in 1 mL PBS containing 50 μM GSNO. The cumulative NO generation was assessed after 2, 6, and 24 h using the Griess assay.

The sustained NO generation of the Se@PDA NPs was evaluated using a free radical analyzer. A NO probe was immersed in a 3.95 mL Se@PDA‐2 suspension until a stable baseline current was reached. Then, 50 μL of GSNO was added to the suspension, resulting in a final concentration of 12.5 μM. The changes in current were recorded over time. To mimic physiological conditions, the recyclability of Se@PDA‐2 NPs was also examined in the presence of a reducing agent glutathione (GSH). Briefly, a NO probe was immersed in a 3.95 mL Se@PDA‐2 suspension containing 1 mM GSH. Then, 50 μL of GSNO was added to the suspension and the changes in current were recorded over time. GSNO was added four additional times after the current signal of each cycle was stable. The experiments were conducted at 37 °C, protected from light, and under constant stirring (400 RPM). The catalytic efficiency was calculated based on the following equation:

(3) $$\text{Catalytic efficiency }\left(\%\right)=\;\frac{A_{i}}{A_{1}}\times 100$$

where *A*_1_ is the average NO_[peak]_ generated from the first GSNO dosage and *A*_i_ is the average NO_[peak]_ generated from the following cycles (*i* = 2nd, 3rd, 4th, and 5th cycles).

##### Statistical Analysis

NO levels, cell viabilities, and fluorescence intensities are presented as mean ± standard deviation. Sample sizes are indicated in the figure captions (*n *= 3 for NO levels, *n *= 5 for cell viabilities, and *n* = 4 for fluorescence intensities). Statistical analyses were performed using GraphPad Prism 10 software. One‐way ANOVA followed by Tukey's post hoc test (α = 0.05) and two‐way ANOVA with Sidak's multiple comparisons test (α = 0.05) were used to assess statistical significance. Significant differences between the groups were expressed as: ns = no significance, *p < 0.05, **p < 0.01, ***p < 0.001, ****p < 0.0001.

## Conflict of Interest

The authors declare no conflict of interest.

## Author Contributions

**Shu Geng**: conceptualization (lead); methodology (lead); investigation (lead); formal analysis (lead); writing—original draft (lead); writing—review & editing (lead). **Qingqing Fan**: conceptualization (supporting); methodology (supporting); investigation (supporting). **Kang Lin**: methodology (supporting); investigation (supporting). **Federico Mazur**: writing—review & editing (supporting). **Rona Chandrawati**: conceptualization (supporting); methodology (supporting); supervision (lead); resources (lead); funding acquisition (lead); writing—review & editing (supporting).

## Supporting information

Supplementary Material

## Data Availability

The data that support the findings of this study are available from the corresponding author upon reasonable request.
