# Supplementary material for: Polydopamine‐Coated Selenium Nanoparticles as a Stable Catalyst for Tunable and Sustained Nitric Oxide Generation
Source: Small Sci. 2025 Jun 1;5(8):2500151. doi: 10.1002/smsc.202500151 (PMC12362805; doi:10.1002/smsc.202500151)
Supplement: Supplementary file 1 — Supplementary Material [file SMSC-5-2500151-s001.pdf]

Supporting Information

Polydopamine-coated selenium nanoparticles as a stable catalyst for tunable and sustained nitric oxide generation

Shu Geng,[a] Qingqing Fan,[a] Kang Lin,[a] Federico Mazur,[a] and Rona Chandrawati[a,*]

[a] School of Chemical Engineering and Australian Centre for Nanomedicine (ACN), The University of New South Wales (UNSW Sydney), Sydney, NSW 2052, Australia

E-mail: rona.chandrawati@unsw.edu.au

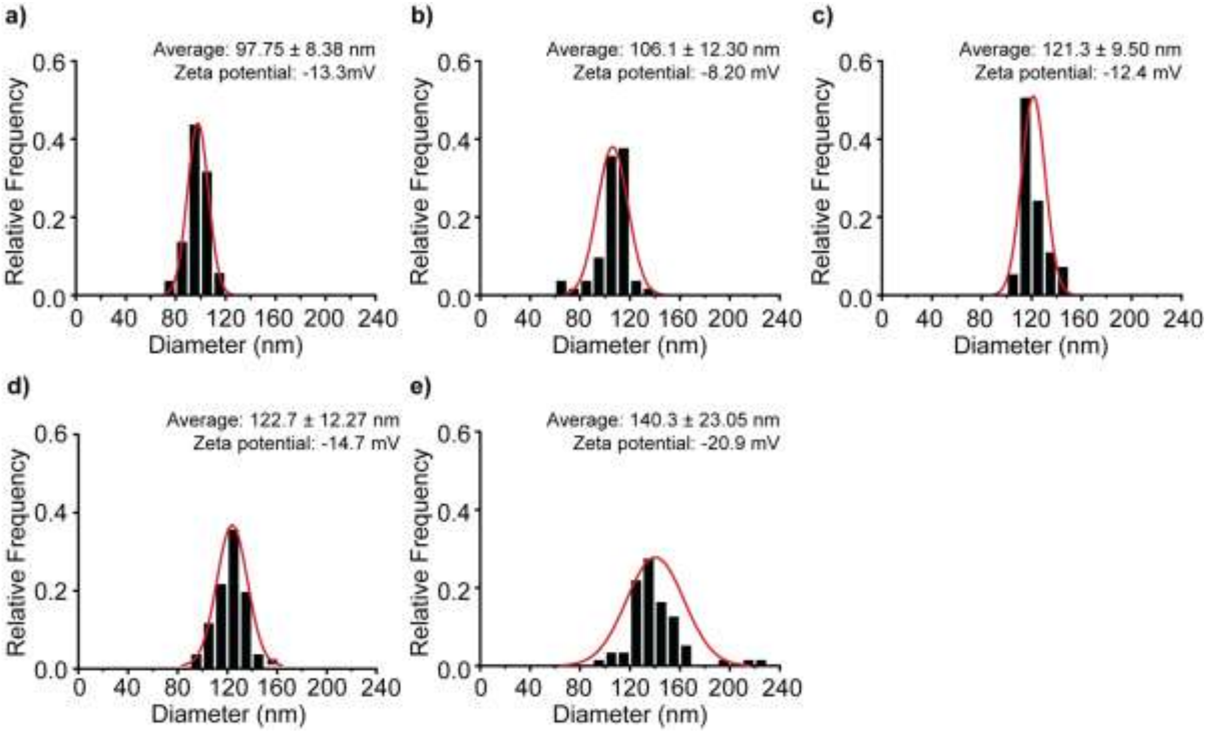

Figure S1. Size distribution and zeta potential of a) SeNPs, b) Se@PDA-2 NPs, c) Se@PDA-4 NPs, d) Se@PDA-6 NPs, and e) Se@PDA-12 NPs.

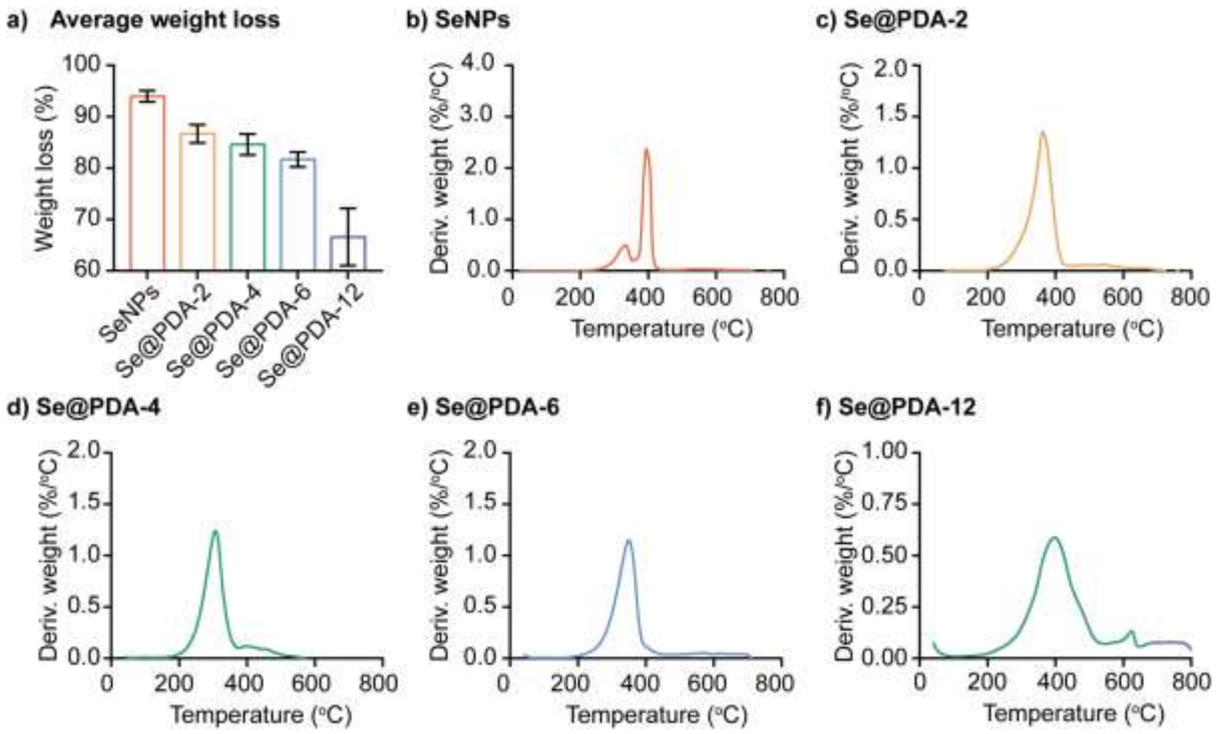

Figure S2. a) Average weight loss at the 400 °C breakpoint. The differential thermogravimetry (DTG) of b) SeNPs, c) Se@PDA-2 NPs, d) Se@PDA-4 NPs, e) Se@PDA-6 NPs, and f) Se@PDA-12 NPs.

Table S1. The corresponding peak positions, peak intensity, and the intensity ratio of SeNPs and Se@PDA NPs.

	**Se shift (cm^{-1})**	**G band shift (cm^{-1})**	**Intensity (I_{Se})**	**Intensity (I_G)**	**I_G/I_{Se}**
SeNPs	233.94	1587.08	5314.22	1937.42	0.36
Se@PDA-2 NPs	240.54	1569.74	1597.54	5236.45	3.28
Se@PDA-4 NPs	245.02	1575.72	1597.75	6709.85	4.20
Se@PDA-6 NPs	247.42	1579.42	4067.24	30498.54	7.50
Se@PDA-12 NPs	249.55	1596.52	4067.57	66119.77	16.26

Table S2. Atomic percentage of O, N, C, and Se in SeNPs and Se@PDA NPs.

	**O1s (%)**	**N1s (%)**	**C1s (%)**	**Se3d5 (%)**
SeNPs	29.7	0	43.75	26.08
Se@PDA-2NPs	28.59	6.7	49.74	14.97
Se@PDA-4 NPs	28.2	6.78	56.92	8.11
Se@PDA-6 NPs	29.18	9.04	57.22	4.56
Se@PDA-12 NPs	29.3	9.99	60.02	0.69

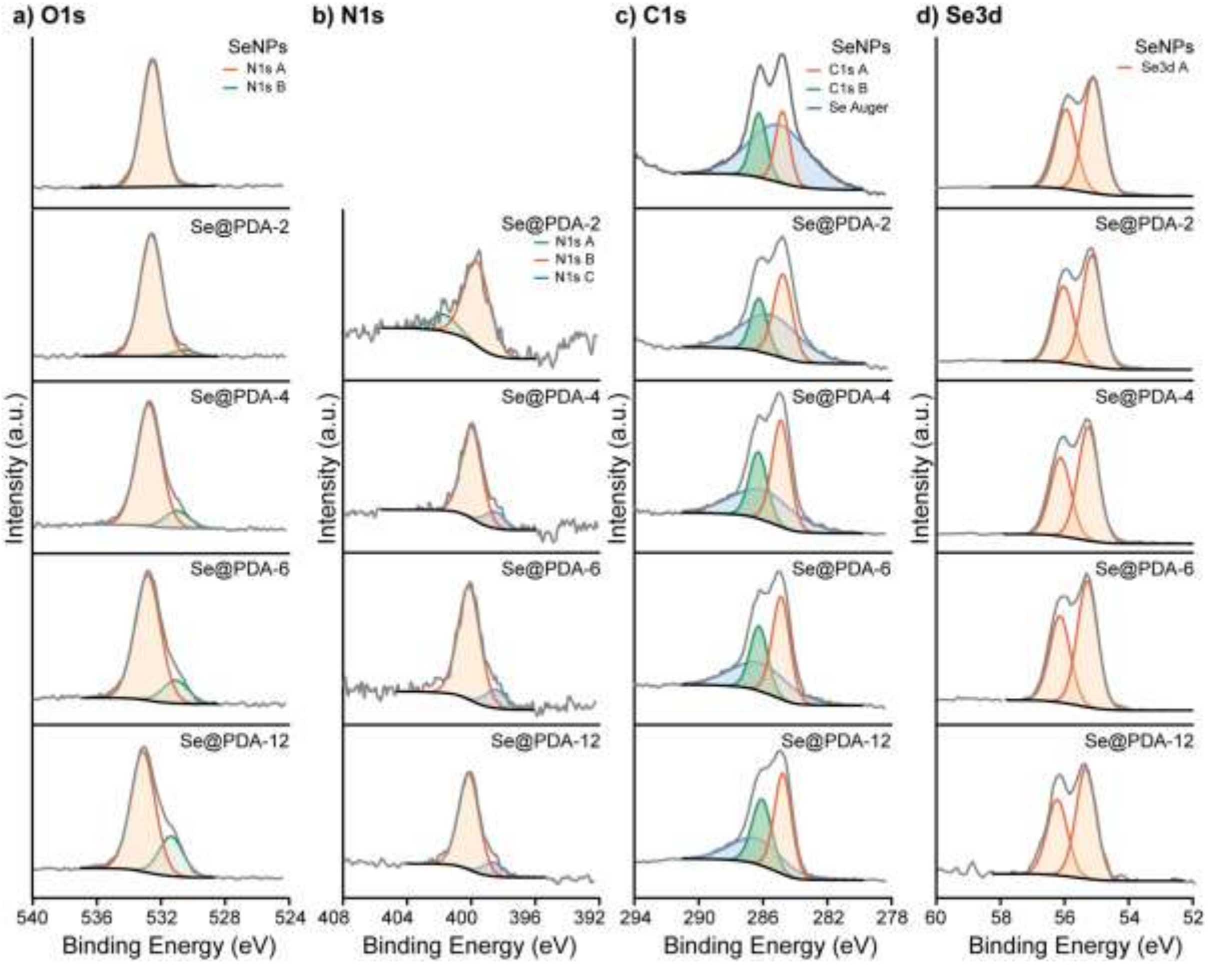

Figure S3. High resolution spectra of a) O1s, b) N1s, c) C1s, and d) Se3d for SeNPs, Se@PDA-2 NPs, Se@PDA-4 NPs, Se@PDA-6 NPs, and Se@PDA-12 NPs.

Table S3. Analyzed XPS peaks and the corresponding chemical bonds.

Peaks		Assigned groups	Binding energy (eV) & Area %				
			**SeNPs**	**Se@PDA-2**	**Se@PDA-4**	**Se@PDA-6**	**Se@PDA-12**
**O1s**	O1s A	C-O-H	532.54 (100.0%)	532.66 eV (95.0%)	532.81 eV (87.6%)	532.88 eV (84.5%)	533.09 eV (75.2%)
	O1s B	C=O	-	530.61 eV (5.0%)	531.05 eV (12.4%)	531.14 (15.5%)	531.35 eV (24.8%)
**N1s**	N1s A	$R-NH_2$ (amine)	-	401.86 eV (16.6%)	-	-	-
	N1s B	R_2-NH (pyrrole)	-	399.84 eV (83.4%)	400.15 eV (84.5%)	400.16 eV (86.0%)	400.22 eV (87.4%)
	N1s C	-N= (imine)	-	-	398.70 eV (15.5%)	398.49 eV (14.0%)	398.71 (12.6%)
**C1s**	C1s A	C-C C=C	284.80 eV (50.8%)	284.80 eV (64.6%)	284.80 eV (61.2%)	284.80 eV (61.4%)	284.80 eV (57.0%)
	C1s B	C-O C-N	286.27 eV (49.2%)	286.29 eV (35.4%)	286.21 eV (38.8 %)	286.18 eV (38.6%)	286.12 eV (43.0%)
**Se3d**	$Se3d_{5/2}$	Se(0)	55.09 eV (100.0%)	55.10 eV (100.0%)	55.25 eV (100.0%)	55.27 eV (100.0%)	55.38 eV (100.0%)

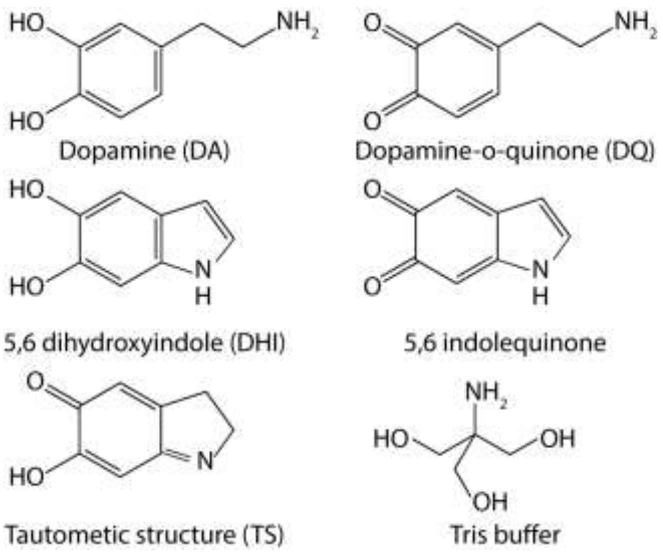

Figure S4. Chemical structures of the building blocks for PDA.

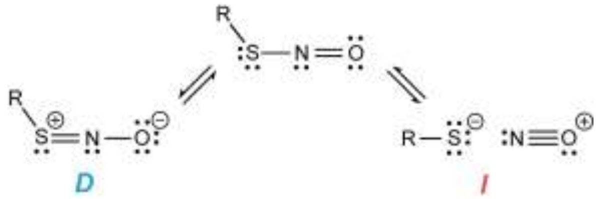

Figure S5. The electron donating (D) and withdrawing (I) resonance electronic structures of GSNO.

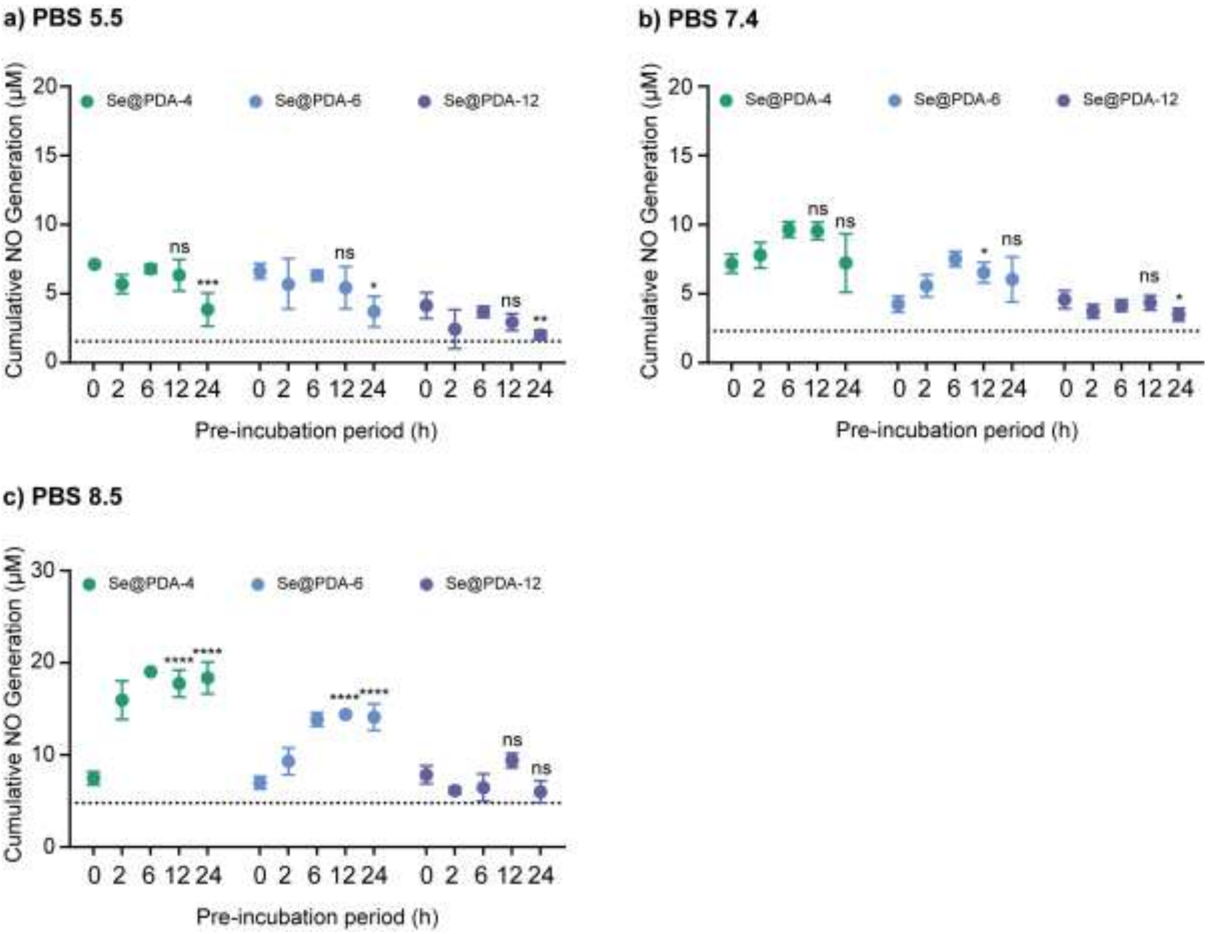

*Figure S6. 4-h cumulative NO generation from GSNO (50 μM) catalysed by 40 μg mL^{-1} of Se@PDA-4 NPs, Se@PDA-6 NPs, or Se@PDA-12 NPs after incubating in PBS buffer at pH a) 5.5, b) 7.4, and c) 8.5, for 0, 2, 6, and 12 h. The dashed line represents the control (GSNO) in corresponding conditions. Statistical significance was calculated using one-way ANOVA, ns = no significance, *p < 0.05, ****p < 0.0001. n=3; error bars represent standard deviation.*

Table S4. The corresponding peak positions, peak intensity and the intensity ratio of Se@PDA-6 NPs before and after pre-incubation in PBS at pH 5.5, 7.4, and 8.5.

	**Se shift (cm^{-1})**	**G band shift (cm^{-1})**	**Intensity (I_{Se})**	**Intensity (I_G)**	**I_G/I_{Se}**
Se@PDA-6	247.42	1579.42	4067.24	30498.54	7.50
Se@PDA-6 in PBS 5.5	235.60	1575.60	4721.89	8574.7	1.82
Se@PDA-6 in PBS 7.4	235.59	1585.31	6279.11	7055.94	1.12
Se@PDA-6 in PBS 8.5	235.59	1578.84	7631.46	5975.05	0.78

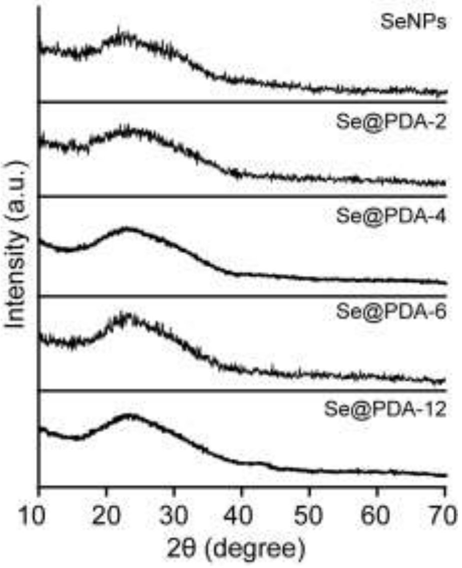

Figure S7. XRD of pristine SeNPs and Se@PDA NPs before reaction

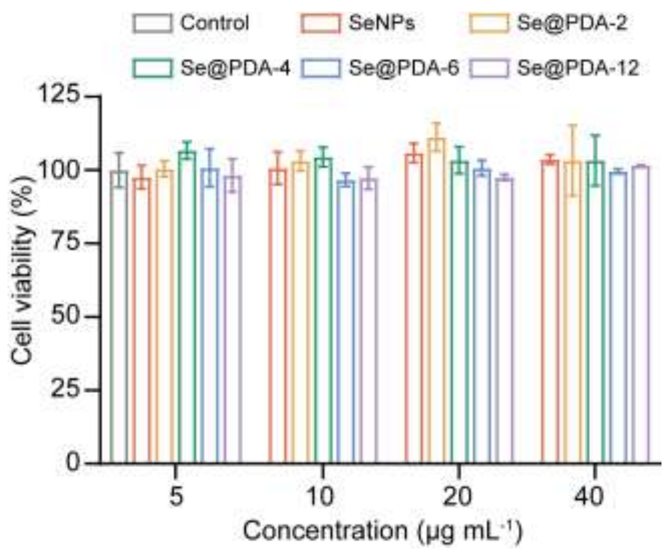

Figure S8. Cell viability of NIH 3T3 cells after 48 h incubation with SeNPs or Se@PDA NPs at various concentrations (5, 10, 20, 40 μg mL^{-1}), assessed using the AlamarBlue assay.

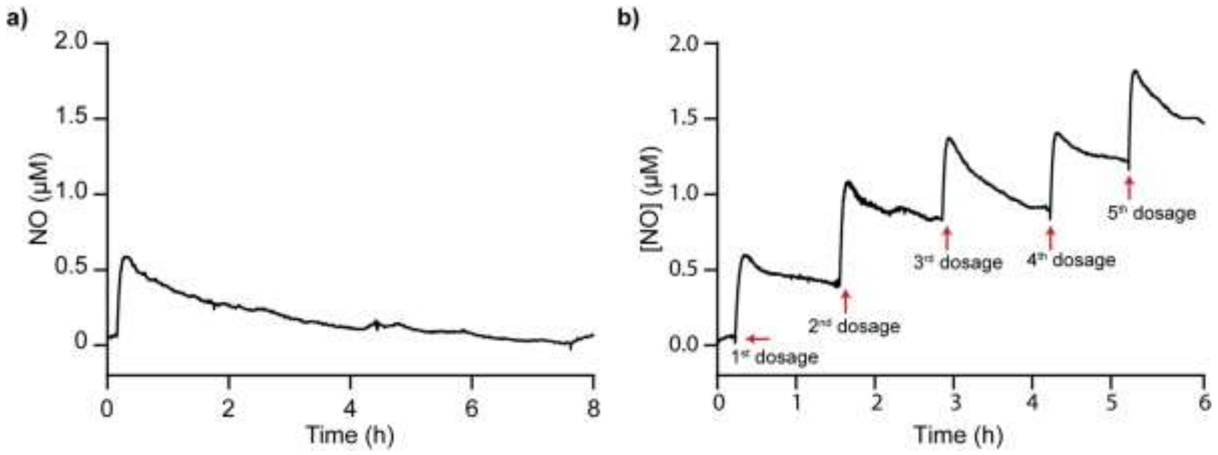

Figure S9. a) Real-time NO generation by Se@PDA-2 NPs (40 μg mL^{-1}) at 37°C in PBS pH 7.4 upon a) single and b) five additions of GSNO (12.5 μM).

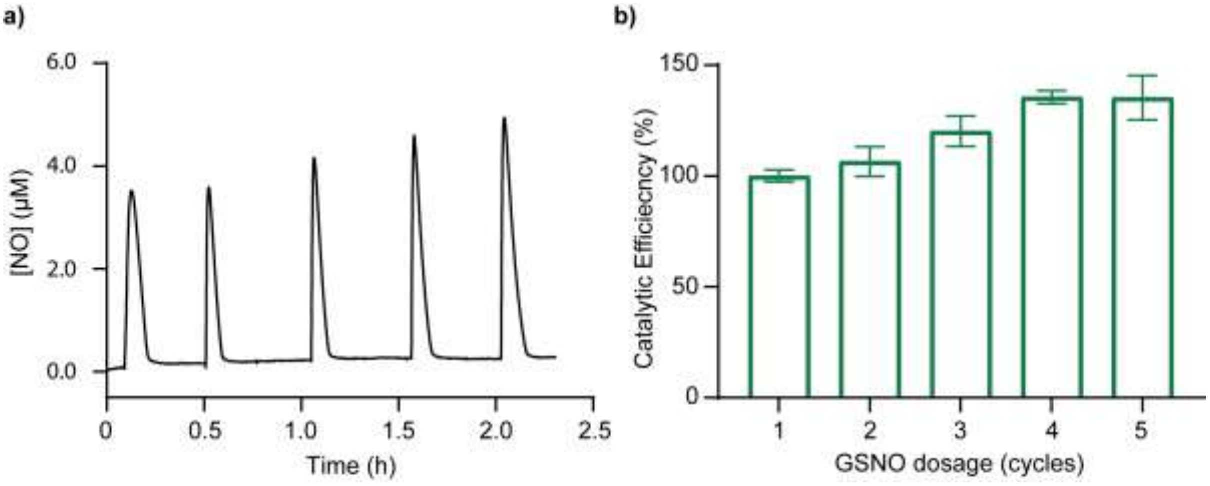

Figure S10. a) Representative sustained NO generation profile and b) calculated catalytic efficiency upon five additions of GSNO (12.5 µM) to a Se@PDA-2 NPs suspension (40 µg mL^{-1}) at 37°C in PBS pH 7.4, in the presence of GSH (1 mM).

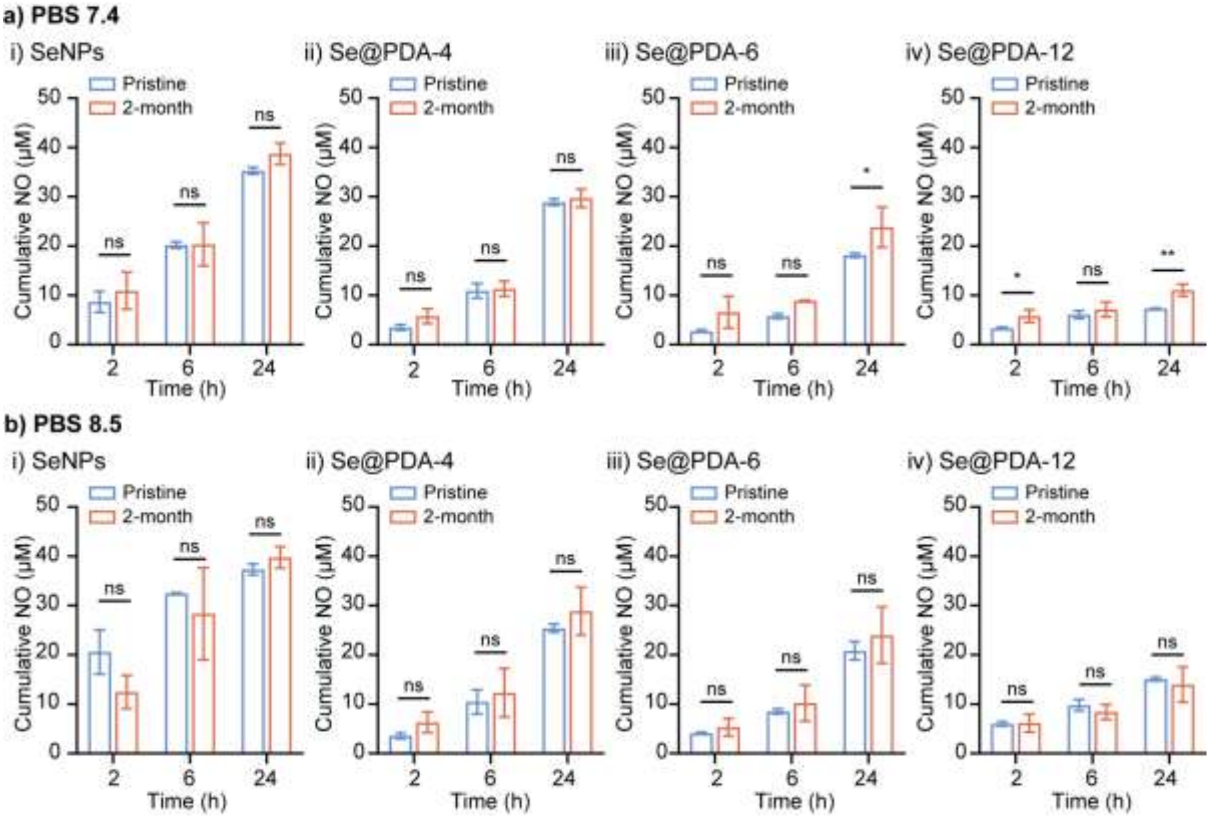

*Figure S11. Cumulative NO generation from GSNO (50 µM) catalysed by 40 µg mL^{-1} of pristine and 2-month stored i) SeNPs, ii) Se@PDA-4 NPs, iii) Se@PDA-6 NPs, and iv) Se@PDA-12 NPs in a) PBS pH 7.4, and b) PBS pH 8.5. Statistical significance was calculated using two-way ANOVA, ns = no significance, *p < 0.05, **p < 0.01. n=3; error bars represent standard deviation.*

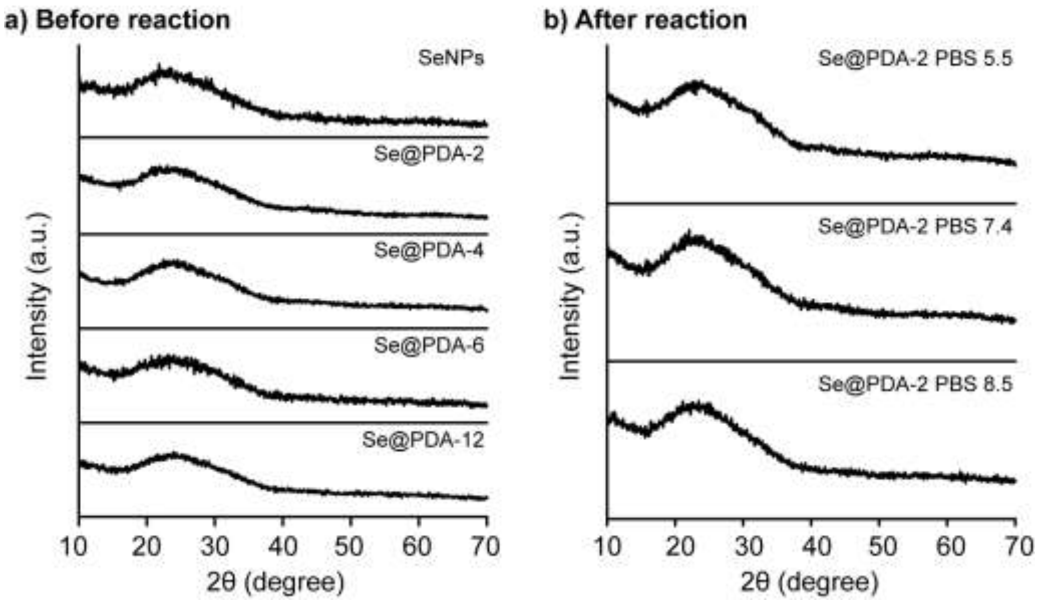

Figure S12. XRD of 2-month stored a) SeNPs and Se@PDA NPs before reaction, and b) Se@PDA-2 NPs after 6 h of incubation with GSNO (50 μM) in PBS buffer at pH 5.5, 7.4, and 8.5.

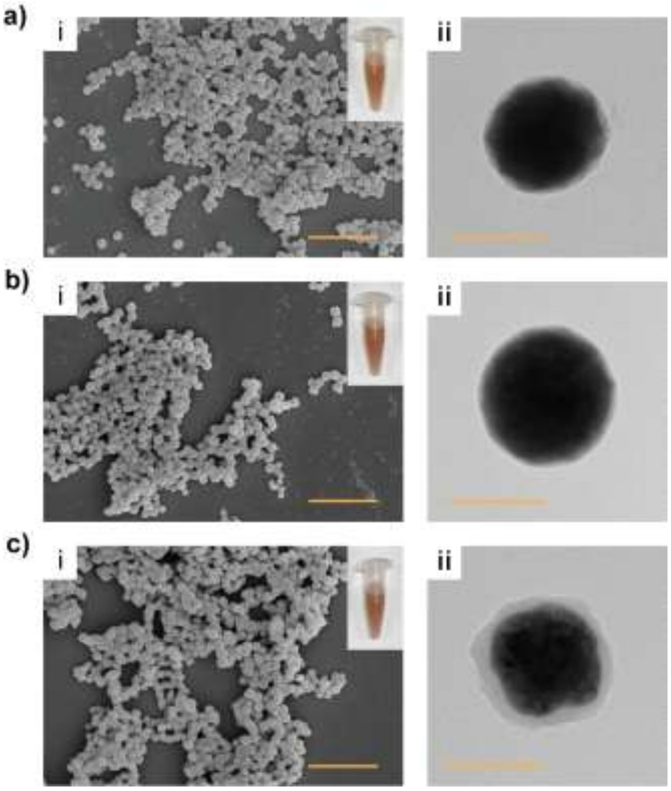

Figure S13. i) SEM and ii) TEM of 2-month stored a) Se@PDA-4 NPs, b) Se@PDA-6 NPs, c) Se@PDA-12 NPs after 8 h of incubation with GSNO (50 μM) and GSH (1 mM) in PBS pH 7.4 (SEM scale bar, 1 μm; TEM scale bar, 100 nm).
